# GLT‐1 downregulation in hippocampal astrocytes induced by type 2 diabetes contributes to postoperative cognitive dysfunction in adult mice

**DOI:** 10.1111/cns.70024

**Published:** 2024-09-01

**Authors:** Xin‐Hao Jiao, Jie Wan, Wei‐Feng Wu, Lin‐Hui Ma, Chen Chen, Wei Dong, Yi‐Qi Liu, Chun‐Hui Jin, Ao Sun, Yue Zhou, Zi‐Yi Li, Qiang Liu, Yu‐Qing Wu, Cheng‐Hua Zhou

**Affiliations:** ^1^ Jiangsu Province Key Laboratory of Anesthesiology, NMPA Key Laboratory for Research and Evaluation of Narcotic and Psychotropic Drugs Xuzhou Medical University Xuzhou China; ^2^ Department of Anesthesiology and Perioperative Medicine, Shanghai Fourth People's Hospital, School of Medicine Tongji University Shanghai China; ^3^ Department of Anesthesiology, National Cancer Center/National Clinical Research Center for Cancer/Cancer Hospital Chinese Academy of Medical Sciences and Peking Union Medical College Beijing China; ^4^ Jiangsu Key Laboratory of New Drug Research and Clinical Pharmacy Xuzhou Medical University Xuzhou China

**Keywords:** apoptosis, GLT‐1, neuronal hyperexcitability, postoperative cognitive dysfunction

## Abstract

**Aims:**

Type 2 diabetes mellitus (T2DM) is related to an increased risk of postoperative cognitive dysfunction (POCD), which may be caused by neuronal hyperexcitability. Astrocyte glutamate transporter 1 (GLT‐1) plays a crucial role in regulating neuron excitability. We investigated if T2DM would magnify the increased neuronal excitability induced by anesthesia/surgery (A/S) and lead to POCD in young adult mice, and if so, determined whether these effects were associated with GLT‐1 expression.

**Methods:**

T2DM model was induced by high fat diet (HFD) and injecting STZ. Then, we evaluated the spatial learning and memory of T2DM mice after A/S with the novel object recognition test (NORT) and object location test (OLT). Western blotting and immunofluorescence were used to analyze the expression levels of GLT‐1 and neuronal excitability. Oxidative stress reaction and neuronal apoptosis were detected with SOD2 expression, MMP level, and Tunel staining. Hippocampal functional synaptic plasticity was assessed with long‐term potentiation (LTP). In the intervention study, we overexpressed hippocampal astrocyte GLT‐1 in GFAP‐Cre mice. Besides, AAV‐Camkllα‐hM4Di‐mCherry was injected to inhibit neuronal hyperexcitability in CA1 region.

**Results:**

Our study found T2DM but not A/S reduced GLT‐1 expression in hippocampal astrocytes. Interestingly, GLT‐1 deficiency alone couldn't lead to cognitive decline, but the downregulation of GLT‐1 in T2DM mice obviously enhanced increased hippocampal glutamatergic neuron excitability induced by A/S. The hyperexcitability caused neuronal apoptosis and cognitive impairment. Overexpression of GLT‐1 rescued postoperative cognitive dysfunction, glutamatergic neuron hyperexcitability, oxidative stress reaction, and apoptosis in hippocampus. Moreover, chemogenetic inhibition of hippocampal glutamatergic neurons reduced oxidative stress and apoptosis and alleviated postoperative cognitive dysfunction.

**Conclusions:**

These findings suggest that the adult mice with type 2 diabetes are at an increased risk of developing POCD, perhaps due to the downregulation of GLT‐1 in hippocampal astrocytes, which enhances increased glutamatergic neuron excitability induced by A/S and leads to oxidative stress reaction, and neuronal apoptosis.

## Graphical abstract

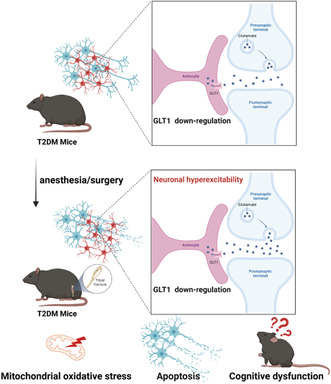

## 1 INTRODUCTION

Postoperative cognitive dysfunction is a serious postoperative complication, characterized by distraction and cognitive impairment.^1^, ^2^ Clinical studies have shown that POCD can gravely affect patients' postoperative recovery and quality of life and increase their dependence on social transfer payments.^3^ However, there is still no effective countermeasure to reduce or prevent POCD. The better understanding of the mechanism behind POCD is necessary. Although the existing clinical and basic exploration of the mechanism of POCD is mainly focused on perioperative stress, neuroinflammation, and synaptic plasticity,^4^, ^5^, ^6^ there is evidence that neuronal hyperexcitability may play a significant role in the occurrence of POCD.^7^ After awakening under general anesthesia, especially under inhalation anesthesia, the excitability of mouse neurons is increased.^8^ This increase in excitability can induce neuronal excitation‐inhibition imbalance, oxidative stress, and even neuronal apoptosis.^9^, ^10^, ^11^ Considering the vulnerability of the aging brain to various stress responses,^12^ postoperative cognitive impairment is most often studied in the elderly.^13^ Nevertheless, POCD shows significant individual differences in the elderly, and POCD also occurs in young adult patients.^14^

Diabetes or insulin resistance increases the risk of POCD, although the relationship remains unclear.^15^ Diabetes is a common chronic metabolic disease characterized by hyperglycemia, which is related to defects in insulin secretion or utilization.^16^, ^17^ In recent years, diabetes has become a global health issue, with type 2 diabetes accounting for the majority.^18^ T2DM is a chronic progressive metabolic disorder that extensively damages organs and tissues, including the central nervous system.^19^, ^20^, ^21^ T2DM can increase the vulnerability of the brain and lead to changes in brain structure and function during its development.^22^ People with T2DM may also need to receive treatment with anesthesia and surgery for conditions not related to or related to diabetes, which increases perioperative risk and risk of complications.^15^, ^23^ An important question is whether individuals with type 2 diabetes are more likely to develop POCD.

The hippocampus is one of the important areas in the brain that is primarily involved in memory and learning processes.^24^The neurotransmitter glutamate plays a key role in the hippocampus, and its transmission between neurons is regulated by the glutamate transporter 1.^25^ GLT‐1 is a glutamate transporter that is mainly expressed in astrocytes, and its function is to recycle glutamate from the synaptic cleft into the interior of astrocytes, preventing excessive accumulation of glutamate around neurons.^26^ By maintaining a stable concentration of glutamate, GLT‐1 not only regulates the excitability of neurons, but also helps protect neurons from excitotoxicity.^27^, ^28^ The abnormal expression of GLT‐1 is closely related to various neurological disorders, including Alzheimer's disease (AD), Parkinson's disease (PD), amyotrophic lateral sclerosis (ALS), epilepsy, and autism.^26^ In addition, GLT‐1 deficiency can enhance susceptibility to excitatory damage, resulting in abnormal behavior and memory impairment.^29^, ^30^, ^31^ When GLT‐1 in hippocampal astrocytes is reduced, the clearance of glutamate in the synaptic cleft decreases, leading to an increase in the concentration of glutamate. Excessive accumulation of glutamate in the synaptic cleft can lead to increased neuronal excitation and induce neuronal apoptosis.^32^ However, it is unclear whether hippocampal astrocyte GLT‐1 participates in the development of POCD in mice with type 2 diabetes.

The purpose of this study was to explore whether type 2 diabetes can facilitate the increased hippocampal neuronal excitability induced by anesthesia/surgery and lead to postoperative cognitive dysfunction, and to determine the key role of astrocyte GLT‐1 in it. Besides, we examined the oxidative stress reaction and neuronal apoptosis due to hyperexcitability.

## 2 MATERIALS AND METHODS

### 2.1 Animals

C57BL/6J mice were purchased from GemPharmatech. Studies involving *GFAP‐Cre* mice (Jackson Laboratories, Stock # 012886) were performed by mating homozygous mice to generate genotyped mice capable of using. The mice were placed in plastic cages (4–5 mice/cage) on a 12 h/12 h light/dark cycle at 23 ± 1°C with free access to water and food. All experiments were approved by the Animal Care and Use Committee of Xuzhou Medical University and conformed to the National Institutes of Health Guide for the Care and Use of Laboratory Animals.

### 2.2 Mouse model of T2DM

C57BL/6 mice (6 weeks old) were fed with a high‐fat diet for 8 weeks. The diabetic model was induced by intraperitoneal injection of streptozotocin (STZ) (Sigma‐Aldrich, S0130; dissolved in 50 mM citric acid buffer) at 40 mg/kg, after a 12 hour fast. The injection was daily for 3 days. Mice with random blood glucose levels above 16.7 mM/L after the last injection were diagnosed with diabetes.^33^ Type 2 diabetic mice were randomly assigned to a type 2 diabetes group (T2DM) or a type 2 diabetes plus anesthesia and surgery group (T2DM + A/S). C57BL/6 mice of the same age were randomly assigned to a control group (Ctrl) or an anesthesia and surgery group (A/S). We used the same approach to establish type 2 diabetes in GFAP‐Cre mice. We also measured the mice's weight and water consumption (Figure S1).

### 2.3 Mouse model of POCD

We performed tibial fracture (TF) with intramedullary fixation under isoflurane anesthesia to establish the POCD model as described in previous studies.^34^ Briefly, mice were anesthetized with 3.0% isoflurane and then maintained with 1.5% isoflurane. Next, we shaved the fur around the knee joint of the mice's left lower extremity and sterilized skin with a 10% povidone‐iodine solution. We then incised the skin on the lateral side of the tibia and exposed the bone. The intramedullary fixation pin was driven to fix the fracture, tibia osteotomy in the middle and lower parts was executed, and the incision was sutured. Last, lidocaine (2%) locally was injected to prevent postoperative pain. During the anesthesia, we used heating pad to maintain the body temperature of the mice between 36°C and 37°C. All mice were sent back to the cages after they recovered from anesthesia. Mice from A/S group and T2DM + A/S group received the above operation.

### 2.4 Stereotaxic surgery and microinjections

To overexpress GLT‐1, we performed stereotactic injection of rAAV‐Efla‐DIO‐GLT1‐EGFP‐WPRE‐pA (BrainVTA) into the hippocampal CA1 region of GFAP‐Cre mice fed with HFD 4 weeks before the surgery. The mice were assigned to a GLT‐1 group, after confirming that type 2 diabetes model was established successfully. As a control, we injected rAAV‐Efla‐DIO‐EGFP (BrainVTA) into the hippocampal CA1 region of GFAP‐Cre mice fed with HFD 4 weeks before the surgery and the mice were assigned to a EGFP group, after confirming that type 2 diabetes model was established successfully. Similarly, to chemogenetically manipulated the activity of Camkllα positive neurons in hippocampal CA1, we injected pAAV‐Camkllα‐hM4D(Gi)‐mCherry‐3xFLAG‐WPRE (Obio Tech) into the hippocampal CA1 region of C57BL/6 mice fed with HFD 4 weeks before the surgery. The mice were assigned to a hM4Di + CNO group, after confirming that type 2 diabetes model was established successfully. As a control, we injected pAAV‐Camkllα‐mCherry‐3xFLAG‐WPRE, (Obio Tech) into the CA1 region of C57BL/6 mice fed with HFD 4 weeks before the surgery. The mice were assigned to a mCherry+CNO group, after confirming that type 2 diabetes model was established successfully. For chemogenetic manipulation of neurons, a single intraperitoneal injection of clozapine‐N‐oxide (CNO, 2.5 mg/kg, dissolved in DMSO, HY‐17366, MCE) was performed 30 min prior to tibial fracture.

Mice received the injection of 1% pentobarbital sodium for anesthesia (40 mg/kg) and fixed in a small animal stereotactic apparatus (RWD) with heating pad maintaining the body temperature of the mice. We applied erythromycin eye ointment to the eyes to prevent conjunctival infection. After sterilization, craniotomy was performed to expose the bregma point of the skull. The virus was microinjected in the CA1 region (anterior–posterior, −1.94 mm; lateral‐medial, ±1.2 mm; dorsal‐ventral, −1.55 mm). The virus was injected bidirectionally for 10 min at a rate of 20 nL/min using a 33‐gauge syringe needle (Hamilton, 65,460–02) and a pump (Harvard Apparatus). Then, we waited 10 min to allow the viral vector to diffuse away and slowly withdrew the needle followed by suturing scalp. In addition, all mice subjected to virus microinjection were exposed to type 2 diabetes and received A/S 28 days after microinjection.

### 2.5 Behavioral tests

Referring to previous research,^35^, ^36^ we used novel object recognition test to evaluate the memory and cognitive function. The experiment was divided into three stages: habituation, training, and testing. In the habituation phase, every mouse was individually placed in the experimental apparatus (50 cm in length, 50 cm in width, and 40 cm in height) without any object for 10 min. The next day, the mice were exposed for 15 min to the apparatus containing two objects at two corners of the same side (training phase). After 24 h, mice were sent back to the experimental apparatus with one of the familiar objects in each pair replaced with a novel object and every mouse was allowed to explore the arena for 5 min (test phase). OLT was performed to examine the mice's capacity for spatial memory. The experiment was also divided into three stages, but the two original objects were placed at the two diagonal corners instead of being replaced in test phase. 75% ethanol was used to clean experimental site and objects before each mouse exploration, which excluded the effect of olfactory cue. To compare the proportion of time spent exploring a novel object or object in a new location in different groups, the percentage spent exploring one object relative to exploring two objects was calculated.

### 2.6 Western blot analysis

We uniformized the hippocampal tissue by RIPA lysis buffer (Beyotime) containing PMSF. Then the concentration of protein in hippocampus was measured by the bicinchoninic acid (BCA) Protein Assay Kit and calibrated with RIPA lysis buffer. We separated the proteins using SDS‐PAGE and then transferred the proteins to PVDF membranes (Merck Millipore, ISEQ00010). The bands were closed through 5% nonfat milk for 2 h and subsequently incubated in primary antibodies at 4°C overnight. The next day, membranes were incubated with horseradish peroxidase‐binding antibodies (1:2000, Beyotime). The primary antibodies contained anti‐GLT1 (1:2000, 20848S, Cell Signaling Technology), anti‐GAPDH (1:2000, AC001, ABclonal), anti‐SOD2 (1:2000, 13141S, Cell Signaling Technology), and anti‐β‐actin (1:2000, AC004, ABclonal). ECL detection equipment (Beyotime) was used to visualize protein and photograph outcomes. Finally, we quantified the protein with ImageJ software. The original, uncropped image of each cropped blot appearing in File S1.

### 2.7 Immunohistochemistry

We used a frozen microtome (CM1950, Leica) to cut the mice brain into 30 μm coronal sections and continuously collected throughout the hippocampus. 10% goat serum with 0.8% PBST was used to rupture membranes and block the brain sections. The brain sections were incubated with primary antibodies overnight at 4°C, and bound by fluorescent secondary antibodies (1:400, ab150080, ab150113, ab150115, Abcam) for 1 h at 37°C. Finally, we use mounting fluid containing DAPI (ab104139, Abcam) to mount the slides. The staining outcomes were recorded by a confocal microscope (FV1000, Olympus). Primary antibodies included anti‐GLT1 (1:100, 20848S, Cell Signaling Technology), anti‐GFAP (1:200, 3670S, Cell Signaling Technology), anti‐Camkllα (1:400, 50049S, Cell Signaling Technology), anti‐GAD67 (1:400, ab26116, Abcam), and anti‐cFos (1:400, 2250S, Cell Signaling Technology).

### 2.8 Transmission electron microscopy

The mice were sacrificed with deep anesthesia, and the CA1 of the hippocampal was separated. The tissues were immediately cut into 1 mm^3^ size on ice, and then fixed with 2.5% glutaraldehyde overnight at 4°C. These sections were fixed in 1% osmium tetroxide, stained with 2% aqueous solution of uranyl acetate, and dehydrated with gradients of ethanol and acetone. Lastly, the tissues were embedded in epoxy resin. The tissues were cut into sections (70 nm) through Ultramicrotome ((UC7rt) A‐1170). The sections were placed on a copper grid, and stained with 4% uranyl acetate with lead citrate. We used transmission electron microscope (Tecnai G2S pirit Twin) to observe sections and record results.^37^

### 2.9 Hippocampal long‐term potentiation (LTP)

The procedure was performed as described previously.^38^ Mice were decapitated under deep anesthesia. The mice brain was rapidly extracted and 300 μm coronal sections containing hippocampus were cut by Leica VT1000s (Leica Biosystems) vibrating microtome in the cutting solution. fEPSPs were recorded with glass patch positioned in CA1 pipettes that were filled with ACSF. At the same time, the concentric electrode CBARB75 (FHC Inc.) stimulated the Schaffer collateral fibers. The intensity of a single baseline stimulus was approximately 50% of the maximum amplitude at 0.05 Hz. Theta burst stimulation (TBS) induced LTP (consisted of 12 bursts of four pulses at 100 Hz, delivered at an interburst interval of 200 ms). We compared the mean fEPSP slope during the last 20 min of the recording period to the mean fEPSP slope before stimulation to quantify LTP.

### 2.10 Determination of mitochondrial membrane potential (MMP)

MMP was measured by JC‐1 dye (Beyotime). We took out mouse hippocampal tissue. After washing with PBS, the tissue was cut into fragments with scissors. We added pre‐chilled mitochondrial isolation reagent A to the tissue and homogenized it at low temperature. The mitochondria was isolated through differential centrifugation and kept in a storage solution with phenylmethylsulfonyl fluoride. Then, we added JC‐1 staining working solution to the purified mitochondria. Varioskan LUX microplate reader was used to determine the red (590 nm) and green (525 nm) fluorescence intensities of the samples.

### 2.11 TUNEL assay

TUNEL assay (Promega, G3250) was executed on brain sections to evaluate neuronal apoptosis in the hippocampus. We immersed the frozen sections in 4% paraformaldehyde and fixed them at room temperature for 30 min. Next, the entire sample area was covered with Proteinase K (20 μg/mL) at room temperature for 10 min. Finally, we added TUNEL solution to the sample and incubated it at 37°C in the dark for 60 min. In order to ensure uniform coverage of the detection solution and prevent evaporation, sealing films were lightly covered on sample. Confocal microscope was used to observe and capture the outcomes. The proportion of TUNEL positive cells was recorded.

### 2.12 Statistical analysis

The data were analyzed by GraphPad Prism 8.0 (GraphPad Software, Inc.). All data followed a normal variable distribution, which was checked by the Shapiro–Wilk test. The results were shown as the mean ± SEM. The unpaired t test was used to contrast the differences between the two groups. To evaluate the differences among the four groups, one‐way ANOVA was performed. Two‐way ANOVA followed by Tukey's multiple‐comparison test was used to compare how two factors affected numeric results. The threshold for significance was set at *p* < 0.05.

## 3 RESULTS

### 3.1 Adult T2DM mice exhibit cognitive dysfunction and LTP attenuation after anesthesia/surgery

**FIGURE 1:**
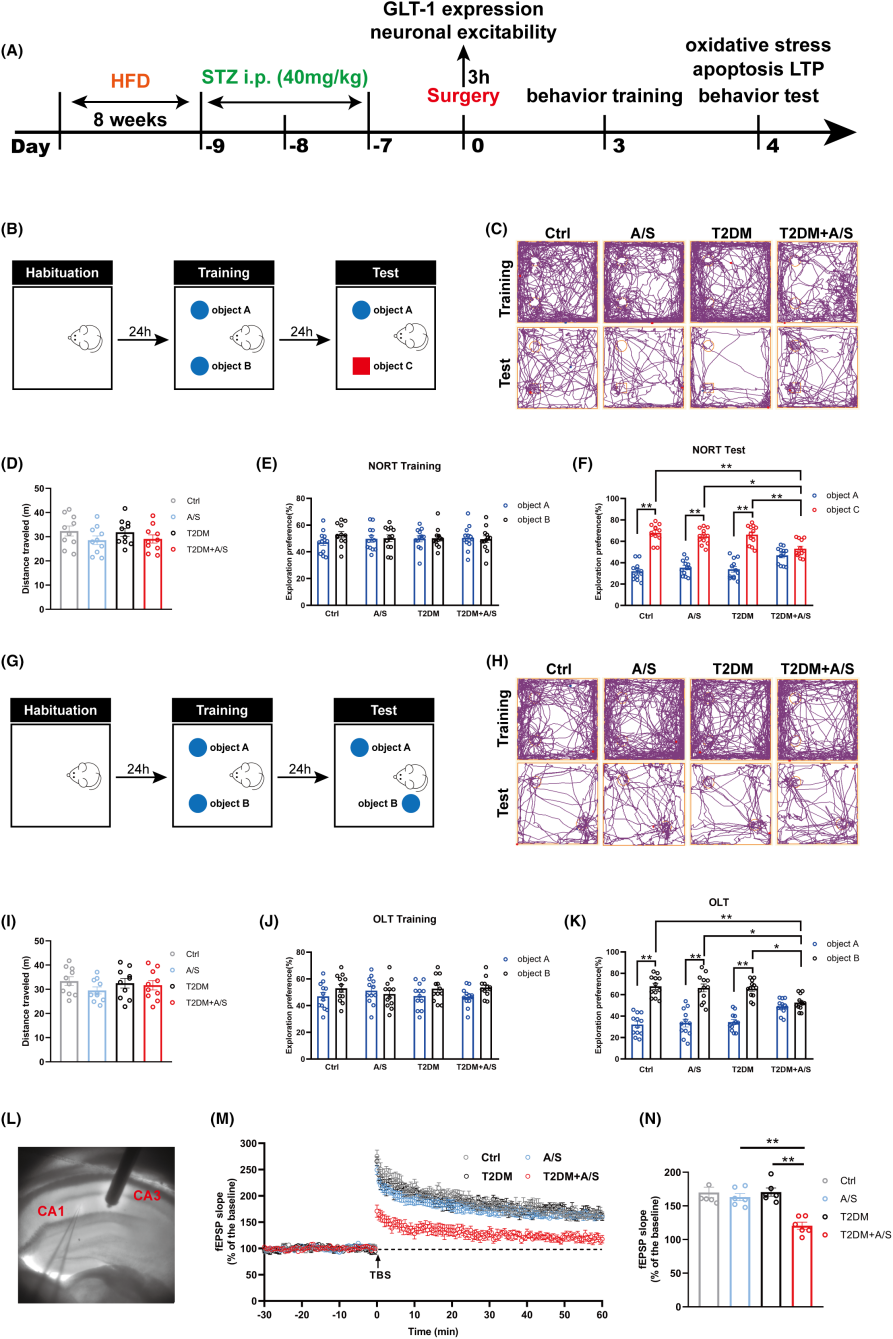
Adult T2DM mice exhibit cognitive dysfunction and LTP attenuation after anesthesia/surgery. (A) Timeline of the experiments. (B) The behavioral paradigm of novel object recognition test (NORT). (C) Representative track diagrams in the NORT. (D) The total movement distance in the habituation period of NORT (*n* = 10). (E) The exploration preference in NORT training (*n* = 10). (F) The exploration preference in NORT test (*n* = 10). (G) The behavioral paradigm of object location test (OLT). (H) Representative track diagrams in the OLT. (I) The total movement distance in the habituation period of OLT (*n* = 10). (J) The exploration preference in OLT training (*n* = 10). (K) The exploration preference in OLT test (*n* = 10). (L) Sample image showing the location of stimulation in the Schaffer collateral and recording in hippocampus CA1 region. (M) LTP recording in the hippocampal CA1 region. The arrow indicated the time point of TBS application. (N) The average fEPSP slope during the last 20 min after TBS (*n* = 6). All data are expressed as the mean ± SEM. *p < 0.05, **p < 0.01.

In the study, we performed anesthesia/surgery on T2DM mice and observed the postoperative cognitive function. The experimental time line is shown in Figure 1A. To elucidate the effect of T2DM on spatial learning and memory after surgery in adult mice, we conducted novel object recognition test and object location test 3 days after anesthesia/surgery (Figure 1B,G). During the habituation phase of the NORT and OLT, there was no statistical difference in the total distance traveled by the mice among the four groups (Figure 1D,I), suggesting the locomotor ability of the mice was normal. The results demonstrated that the percentage of exploration time spent in the two objects of each group was similar during the training phase (Figure 1E,J). During the testing phase, the percentage of exploration time for the new object in the NORT in mice of the T2DM + A/S group was less than that in the A/S group and T2DM group (Figure 1C,F). Consistently, the percentage of time the T2DM + A/S group spent exploring the object in novel location in OLT was reduced compared with the A/S group and T2DM group (Figure 1H,K).

LTP associated with synaptic plasticity and is considered to be a significant measure of learning ability.^39^ We therefore used LTP to record the field excitatory postsynaptic potentials (fEPSP) from the CA1 region following Schaffer collateral stimulation in hippocampal sections from the mice of each group (Figure 1L). The findings showed that theta burst stimulation (TBS)‐induced fEPSP was significantly reduced in the T2DM + A/S group compared to that in A/S group and T2DM group (Figure 1M,N). These results confirmed that adult T2DM mice exhibit cognitive dysfunction and functional synaptic plasticity impairment when suffering anesthesia/surgery.

### 3.2 GLT‐1 expression in hippocampal CA1 astrocytes was downregulated in T2DM mice

**FIGURE 2:**
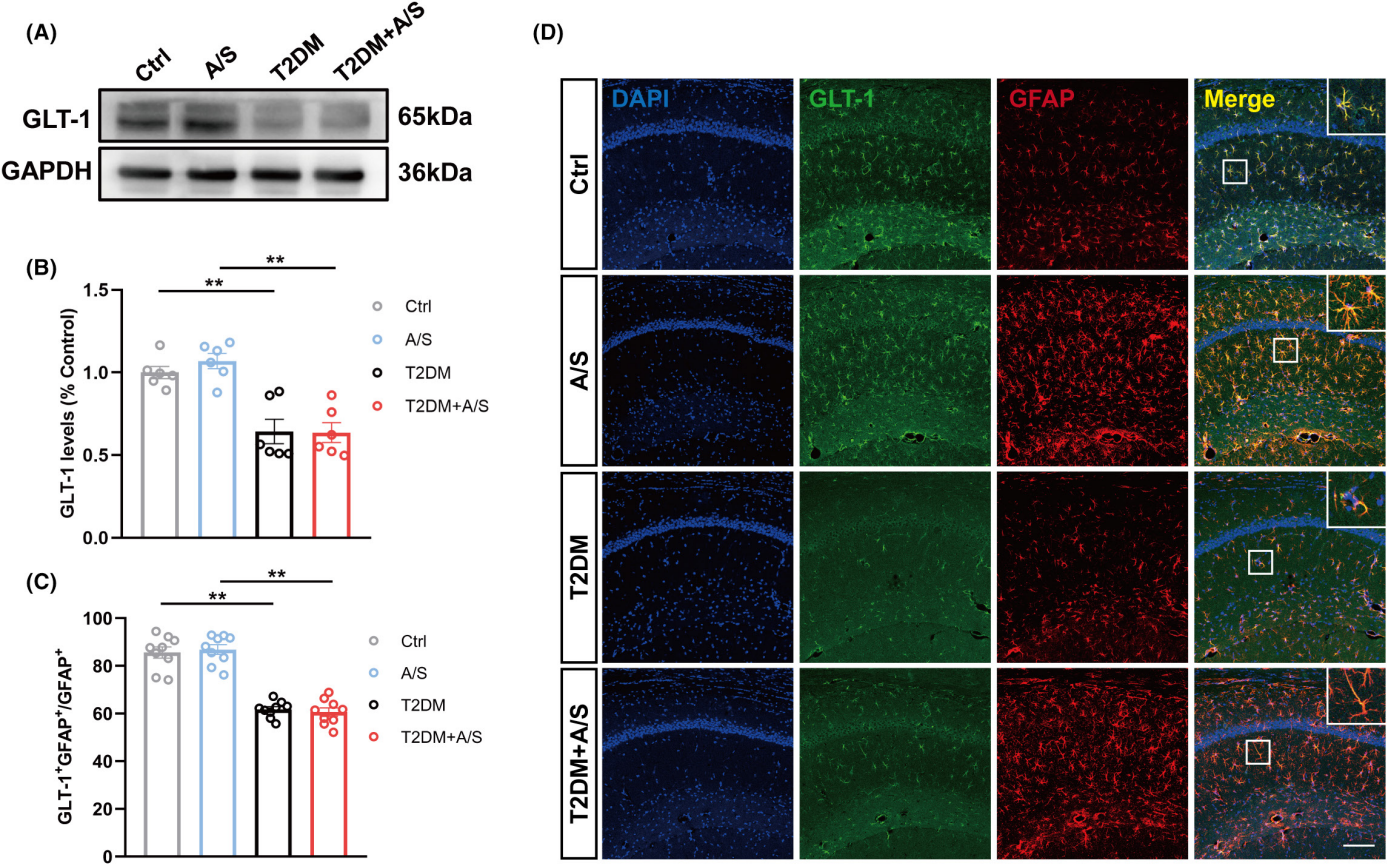
GLT‐1 expression in hippocampal CA1 astrocytes was downregulated in T2DM mice. (A) Representative Western blot of GLT‐1 at 3 h after anesthesia/surgery. (B) Quantitative analysis of GLT‐1 expression compared with the control group (*n* = 6). (C) Quantification of GLT‐1 immunoreactivity in GFAP^+^ cells (*n* = 9). (D) Representative immunofluorescent images of GLT‐1 and GFAP staining at 3 h after anesthesia/surgery. Scale bar: 100 μm. All data are expressed as the mean ± SEM. *p < 0.05, **p < 0.01.

GLT‐1 is mainly localized in astrocytes and plays an important role in cognitive related diseases.^26^ Western blot analysis showed that the expression level of GLT‐1 was significantly decreased in the hippocampus of T2DM mice (Figure 2A,B). In addition, the immunofluorescence outcomes similarly showed a decrease in GLT‐1^+^GFAP^+^/GFAP^+^ in the hippocampus of T2DM group and T2DM + A/S group (Figure 2C,D). Here, we found T2DM but not A/S reduced GLT‐1 expression in hippocampal astrocytes, but this was not sufficient to explain why only mice in T2DM + A/S group developed POCD.

### 3.3 T2DM magnified the increased hippocampal glutamatergic neuron excitability induced by anesthesia/surgery

**FIGURE 3:**
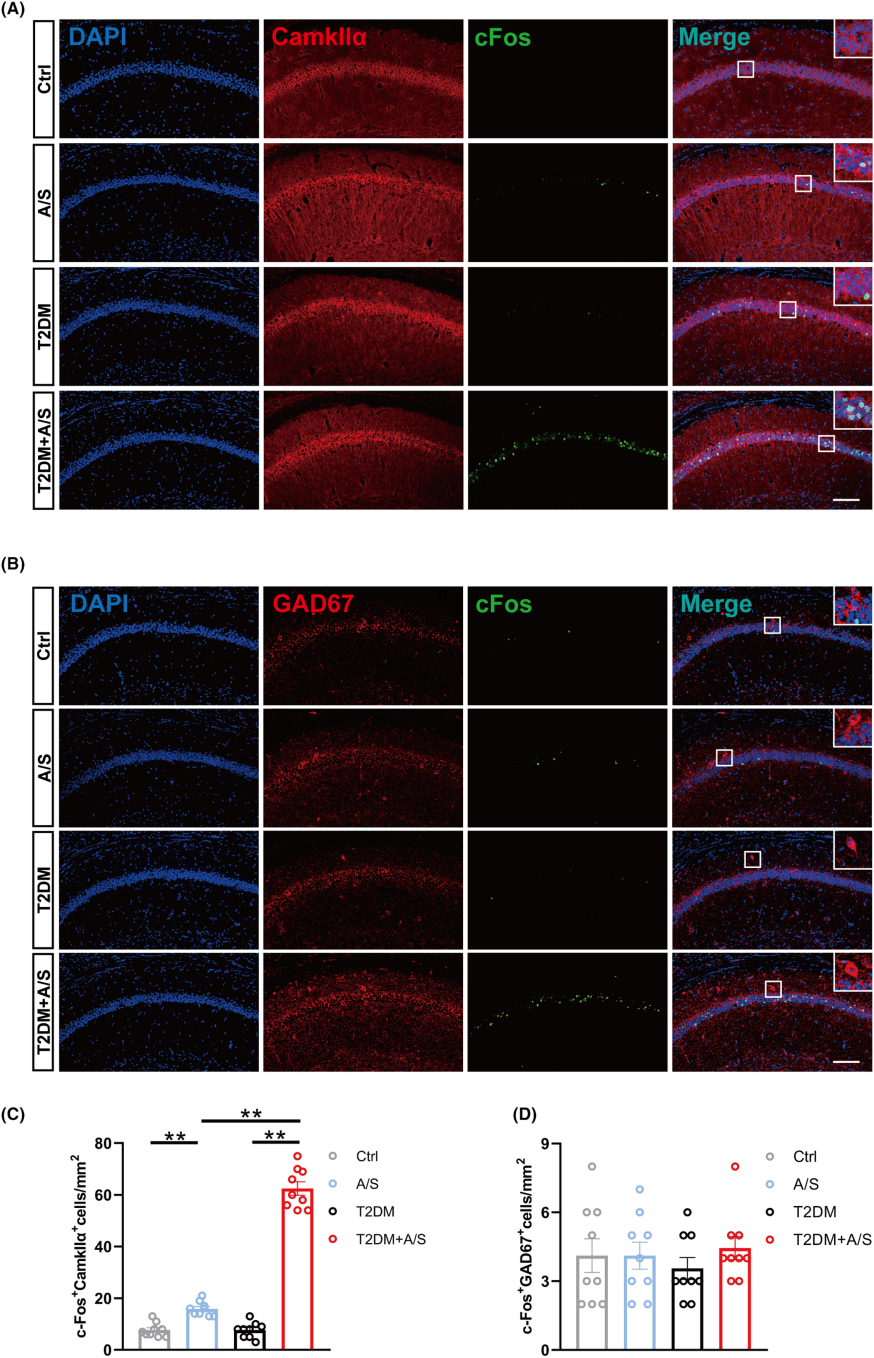
T2DM magnified the increased hippocampal glutamatergic neuron excitability induced by anesthesia/surgery. (A) Representative immunofluorescent images of c‐Fos and Camkllα staining at 3 h after anesthesia/surgery. Scale bar: 100 μm. (B) Representative immunofluorescent images of c‐Fos and GAD67 staining at 3 h after anesthesia/surgery. Scale bar: 100 μm. (C) Quantification of c‐Fos immunoreactivity in Camkllα^+^ cells (*n* = 9). (D) Quantification of c‐Fos immunoreactivity in GAD67^+^ cells (*n* = 9). All data are expressed as the mean ± SEM. *p < 0.05, **p < 0.01.

Previous studies have shown that inhaled anesthetic can activate excitatory neurons. Considering that diabetes altered GLT‐1 expression, which may affect neuronal excitability, we evaluated c‐Fos expression by immunofluorescence staining 3 h after anesthesia/surgery. We counted the number of c‐Fos^+^ cells in glutamatergic neurons or GABAergic neurons in each group (The activated glutamatergic neurons were c‐Fos^+^Camkllα^+^, and the activated GABAergic neurons were c‐Fos^+^GAD67^+^.) The results showed the anesthesia/surgery increased the number of c‐Fos^+^ cells in glutamatergic neurons in both the A/S group and the T2DM + A/S group, but the number of c‐Fos^+^ cells in glutamatergic neurons in T2DM + A/S group was dramatically higher than that in A/S group (Figure 3A,C). However, there was no significant difference in GABAergic neurons (Figure 3B,D). The above results indicated that T2DM promoted anesthesia/surgery‐induced increase in the excitability of hippocampal glutamatergic neurons, and the hyperexcitability could be due to GLT‐1 downregulation, which failed to deal with the excitatory stimulation induced by anesthesia/surgery.

### 3.4 T2DM induced oxidative stress reaction and neuronal apoptosis in hippocampus after anesthesia/surgery

**FIGURE 4:**
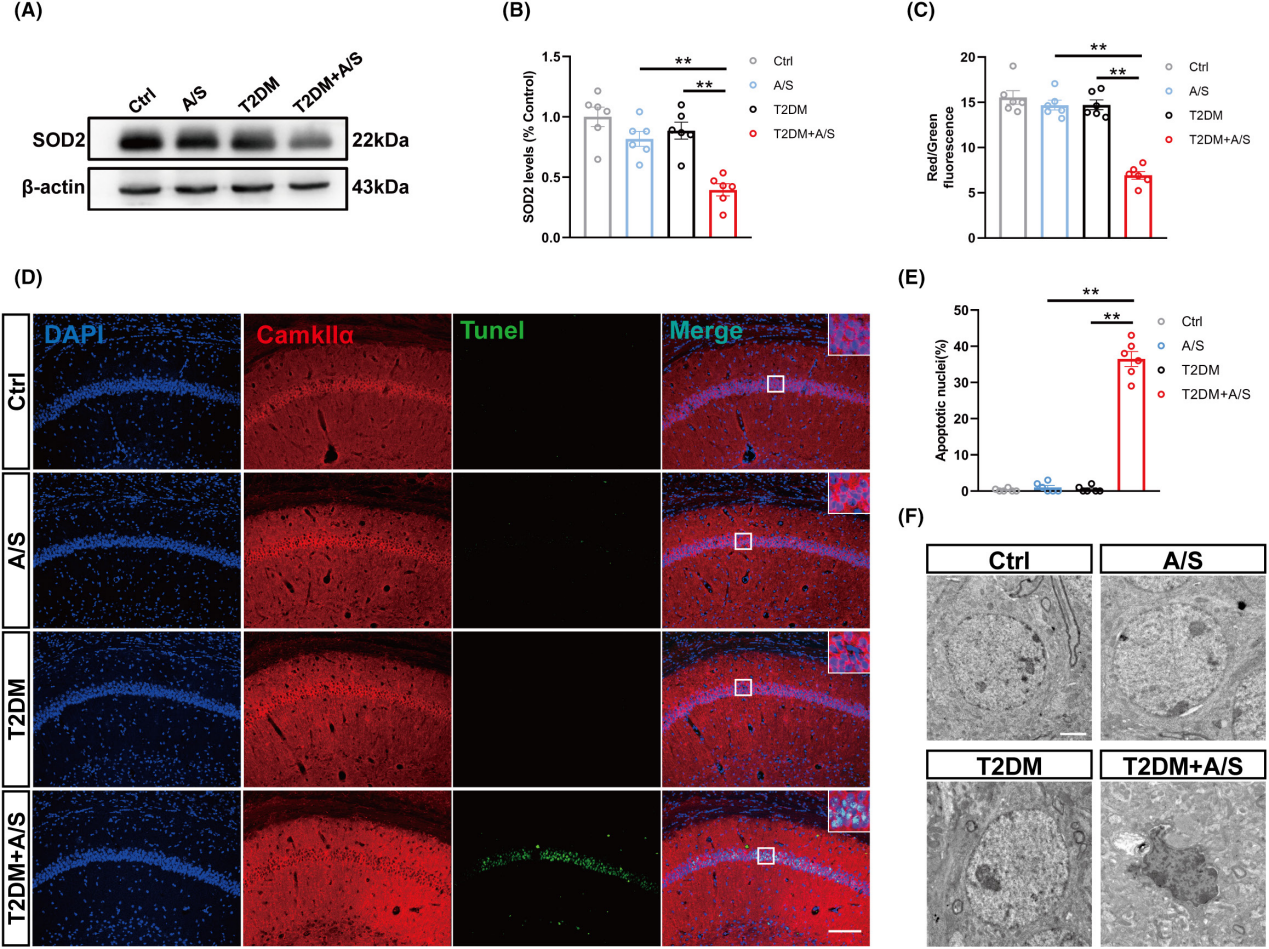
T2DM induced oxidative stress reaction and neuronal apoptosis in hippocampus after anesthesia/surgery. (A) Representative western blot of SOD2 at day 4. (B) Quantitative analysis of SOD2 expression compared with the control group (*n* = 6). (C) Analysis of MMP in the hippocampus region among the four groups at day 4 (*n* = 6). (D) Representative images of Tunel and Camkllα staining at day 4 after anesthesia/surgery. Scale bar: 100 μm. (E) Quantitation of co‐labeling rate of Tunel and Camkllα (*n* = 6). (F) Representative electron micrographs showing nuclear chromatin abnormalities in hippocampal CA1 neurons. Scale bar: 2 μm. All data are expressed as the mean ± SEM. *p < 0.05, **p < 0.01.

The oxidative stress reaction and apoptosis were evaluated to explore the adverse influence of hyperexcitability in glutamatergic neuron of T2DM mice suffering anesthesia/surgery. To evaluate the oxidative stress reaction, we examined SOD2 expression and MMP levels in CA1. The mice in T2DM + A/S group showed markedly decreased in expression of SOD2 and MMP level compared with the mice from the other three groups (Figure 4A–C). Besides, there was an increase in the ratio of terminal deoxynucleotidyl transferase dUTP nick end labeling (TUNEL) positive staining cells in T2DM + A/S group compared to that in the other three groups (Figure 4D,E). Consistently, morphological changes, as observed with transmission electron microscopy, demonstrated that the nuclei within hippocampal CA1 neurons from mice in T2DM + A/S group exhibited remarkable characteristics of apoptosis (Figure 4F).

Considering that neuronal apoptosis and oxidative stress were directly related to cognitive dysfunction, we speculated that the hyperexcitability in hippocampal glutamatergic neuron might be an important cause of POCD, which also explained why POCD only occurred in the mice from the T2DM + A/S group.

### 3.5 GLT1 overexpressed in the hippocampal CA1 astrocytes

**FIGURE 5:**
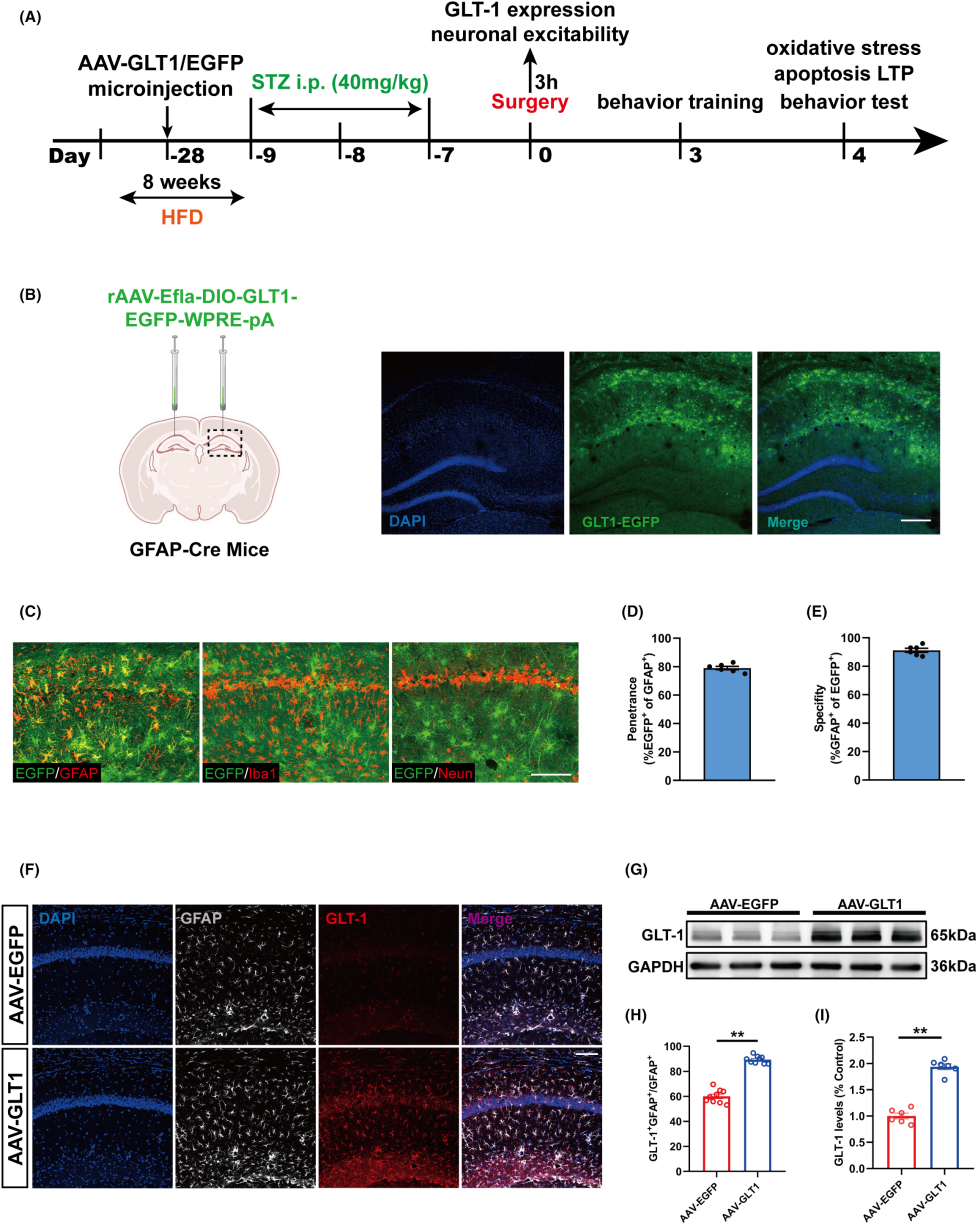
GLT1 overexpressed in the hippocampal CA1 astrocytes. (A) Timeline of the experiments. (B) The location of viral microinjection with a rAAV‐Ef1a‐DIO‐GLT1‐EGFP‐WPRE‐pA (AAV‐GLT1) or a control rAAV‐Ef1a‐DIO‐EGFP‐WPRE (AAV‐VEH). Fluorescence images showing efficient expression of the AAV‐GLT1 vector in the CA1 region. Scar bar = 200 μm. (C) GLT1 (green) was mainly expressed in astrocytes. No co‐localization with the microglia marker Iba1 or the neuron marker NeuN was detected. Scale bar = 100 μm. (D) Percentage of GFAP cells that were EGFP labeled. (E) Percentage of EGFP+ cells expressing GFAP (*n* = 6). (F) Representative immunofluorescent images of GLT‐1 and GFAP staining. Scar bar = 100 μm. (G) Representative Western blot of GLT‐1. (H) Quantification of GLT‐1 immunoreactivity in GFAP^+^ cells (*n* = 9). (I) Quantitative analysis of GLT‐1 expression compared with the EGFP group (*n* = 6). All data are expressed as the mean ± SEM. *p < 0.05, **p < 0.01.

In order to further investigate the magnifying effect of GLT‐1 downregulation on excitatory stimulation induced by anesthesia/surgery, a Cre‐dependent adeno‐associated virus, AAV‐DIO‐GLT1‐EGFP, was administered into the dorsal hippocampal CA1 region of GFAP‐Cre mice 4 weeks prior to the surgery, and injection of AAV‐DIO‐EGFP was performed as a control. After the virus microinjection, the mice received STZ intraperitoneal injection to induce type 2 diabetes, and we performed anesthesia/surgery on them at Day 0, as shown in the flow chart (Figure 5A). Confocal imaging proved that virus was locally expressed in the hippocampus (Figure 5B). In the virally transduced region, there was almost no co‐localization of the microglial marker Iba1 or the neuronal nuclear marker NeuN with EGFP^+^ cells and EGFP^+^ cells were mainly expressed astrocyte marker GFAP (Figure 5C), with high penetrance (>75% of the GFAP‐positive cells expressed EGFP) (Figure 5D) and almost complete specificity (>95% EGFP‐positive cells were also GFAP‐positive) (Figure 5E). Western blotting analysis revealed that the GLT‐1 level was significantly increased in the GLT‐1 group compared to that in the EGFP group (Figure 5G,I). Consistently, immunofluorescence analysis indicated that the virus increased the expression of GLT‐1 in hippocampus astrocyte in GLT‐1 group (Figure 5F,H).

### 3.6 GLT‐1 overexpression attenuated neuropathological injuries induced by hyperexcitability in hippocampus and improved cognitive function in T2DM mice suffering anesthesia/surgery

**FIGURE 6:**
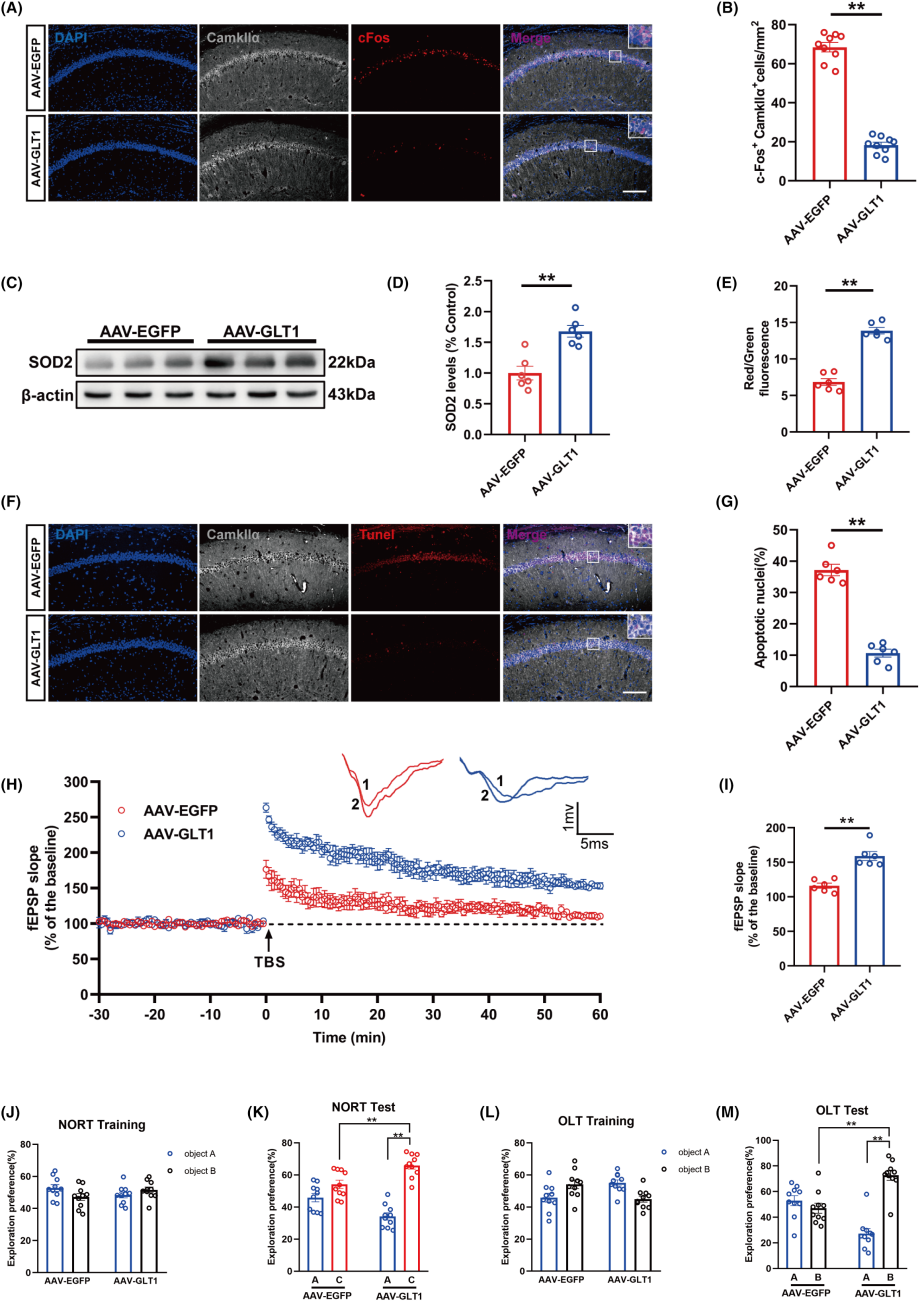
GLT‐1 overexpression attenuated neuropathological injuries induced by hyperexcitability in hippocampus and improved cognitive function in T2DM mice suffering anesthesia/surgery. (A) Representative immunofluorescent images of c‐Fos and Camkllα staining at 3 h after surgery. Scale bar: 100 μm. (B) Quantification of c‐Fos immunoreactivity in Camkllα+ cells (*n* = 9). (C) Representative Western blot of SOD2 at day 4. (D) Quantitative analysis of SOD2 expression compared with the control group (*n* = 6). (E) Analysis of MMP in the hippocampus region among the two groups at day 4 (*n* = 6). (F) Representative images of Tunel and Camkllα staining at day 4 after anesthesia/surgery. Scale bar: 100 μm. (G) Quantitation of co‐labeling rate of Tunel and Camkllα (*n* = 6). (H) LTP recording in the hippocampal CA1 region. The arrow indicated the time point of TBS application. fEPSP slope before TBS was recorded as representative waveform 1 and fEPSP slope after TBS was recorded as representative waveform 2. (I) The average fEPSP slope during the last 20 min after TBS (*n* = 6). (J) The exploration preference in NORT training (*n* = 10). (K) The exploration preference in NORT test (*n* = 10). (l) The exploration preference in OLT training (*n* = 10). (M) The exploration preference in OLT test (*n* = 10). All data are expressed as the mean ± SEM. *p < 0.05, **p < 0.01.

The overexpression of GLT‐1 in hippocampal astrocytes significantly reduced the number of c‐Fos^+^Camkllα^+^ cells and prevented the hyperexcitability of glutamatergic neurons in T2DM mice suffering anesthesia/surgery (Figure 6A,B). Additionally, GLT‐1 overexpression increased the SOD2 expression and MMP level effectively (Figure 6C–E). Tunel staining showed that GLT‐1 overexpression decreased ratio of Tunel positive cells in glutamatergic neurons of T2DM mice receiving anesthesia/surgery (Figure 6F,G). These consequences indicated that GLT‐1 overexpression ameliorated oxidative stress reaction and reduced neuronal apoptosis in T2DM mice suffering anesthesia/surgery.

Regarding functional synaptic plasticity, GLT‐1 overexpression significantly increased the average of fEPSP and improved long‐term potentiation (LTP) in hippocampus of T2DM mice suffering anesthesia/surgery (Figure 6H,I). Moreover, in the NORT and OLT, the mice in GLT‐1 group exhibited a markedly increase in the exploration time percentage spent in novel object or object in novel location compared with original object or location (Figure 6K,M). During the training phase, there was no significant difference for the exploration time percentage toward the two objects (Figure 6J,L).

Collectively, these results revealed that GLT‐1 overexpression in hippocampal astrocytes could reverse the hyperexcitability in hippocampal glutamatergic neuron and alleviate postoperative cognitive dysfunction.

### 3.7 Chemogenetic inhibition of hippocampal CA1 glutamatergic neurons rescued neuropathological injuries in hippocampus and cognitive dysfunction in T2DM mice suffering anesthesia/surgery

**FIGURE 7:**
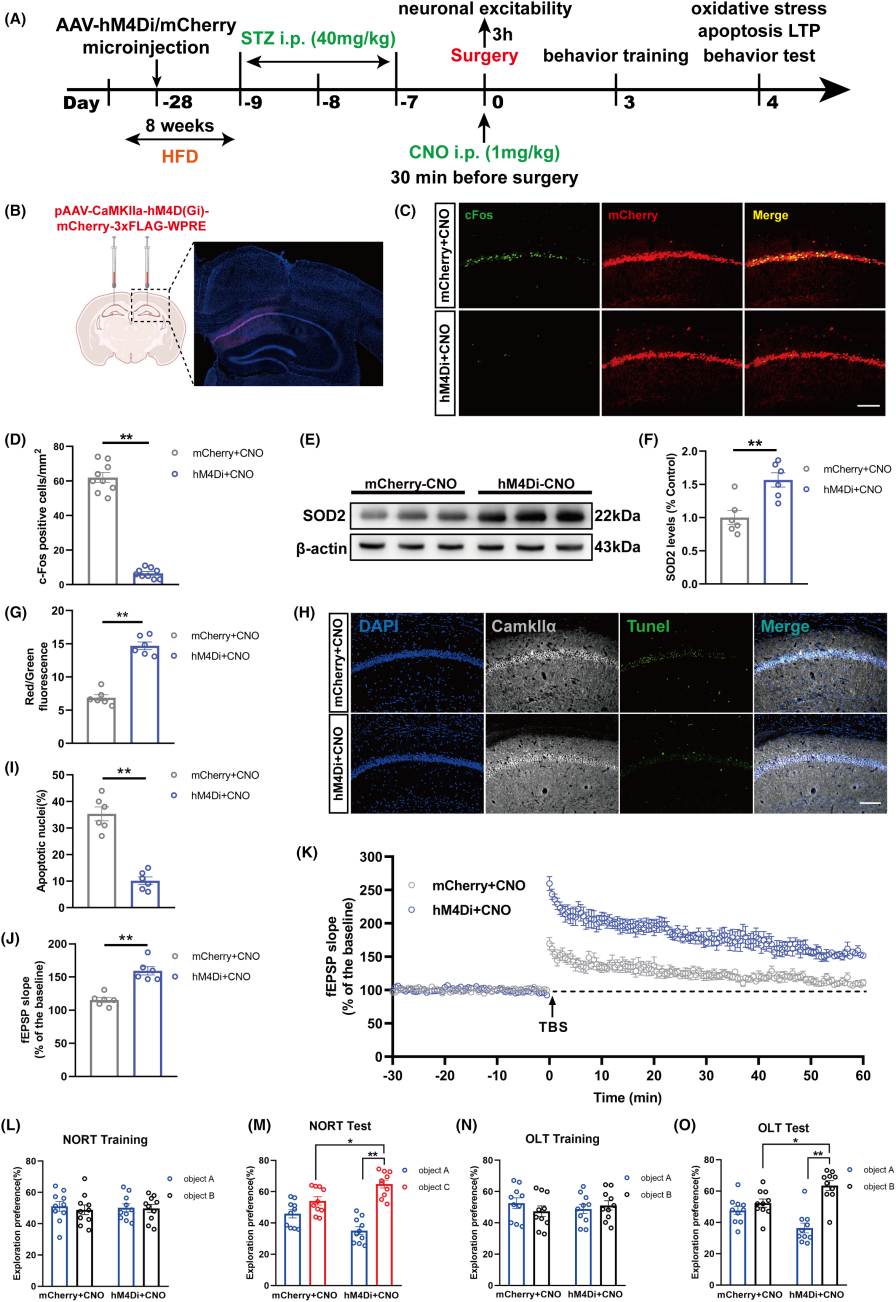
Chemogenetic inhibition of hippocampal CA1 glutamatergic neurons rescued neuropathological injuries in hippocampus and cognitive dysfunction in T2DM mice suffering anesthesia/surgery. (A) Timeline of the experiments. (B) The location of viral microinjection with a pAAV‐Camkllα‐hM4D(Gi)‐mCherry‐3xFLAG‐WPRE (AAV‐hM4Di) or a control pAAV‐Camkllα‐mCherry (AAV‐mCherry). Fluorescence images showing efficient expression of the AAV‐hM4Di in the CA1 region. (C) Representative images of hM4Di‐transduced neurons and c‐Fos expression at 3 h after surgery with intraperitoneal injection of CNO. Scale bar: 100 μm. (D) Quantification of c‐Fos immunoreactivity in mCherry ^+^ cells (*n* = 9). (E) Representative Western blot of SOD2 at day 4. (F) Quantitative analysis of SOD2 expression compared with the control group (*n* = 6). (G) Analysis of MMP in the hippocampus region among the two groups at day 4 (*n* = 6). (H) Representative images of Tunel and Camkllα staining at day 4 after anesthesia/surgery. Scale bar: 100 μm. (I) Quantitation of co‐labeling rate of Tunel and Camkllα (*n* = 6). (J) The average fEPSP slope during the last 20 min after TBS (*n* = 6). (K) LTP recording in the hippocampal CA1 region. The arrow indicated the time point of TBS application. (L) The exploration preference in NORT training (*n* = 10). (M) The exploration preference in NORT test (*n* = 10). (N) The exploration preference in OLT training (*n* = 10). (O) The exploration preference in OLT test (*n* = 10). All data are expressed as the mean ± SEM. *p < 0.05, **p < 0.01.

To determine if hyperexcitability of hippocampal glutamatergic neuron played an important role in POCD of T2DM mice, we expressed the Gi‐coupled receptor hM4Di in CA1 glutamatergic neurons and used a designer drug CNO manipulating its activity chemogenetically. As shown in the flow chart, we injected AAV‐Camkllα‐hM4Di‐mCherry or AAV‐Camkllα‐mCherry into hippocampal CA1 region of mice 4 weeks before surgery. After the mouse model of type 2 diabetes was successfully established, we performed anesthesia/surgery and gave CNO intraperitoneally 30 min before anesthesia/surgery (Figure 7A). Confocal imaging showed that hM4Di was only expressed in the hippocampal CA1 region (Figure 7B). Immunofluorescence staining was used to assess c‐Fos expression in CA1. The results showed that CNO effectively decreased c‐Fos levels in hM4Di^+^ neurons (Figure 7C,D), which suggested that the designer receptors exclusively activated by designer drugs (DREADDs) dramatically inhibited activation of glutamatergic neurons in CA1 region.

Inhibiting the activity of hippocampal CA1 glutamatergic neurons during surgery recovered the SOD2 expression and MMP level (Figure 7E–G). Tunel staining revealed that the percentage of Tunel^+^ Camkllα^+^ cells in hM4Di + CNO group was less than that in mCherry+CNO group (Figure 7H,I). The consequences illustrated DREADDs attenuated oxidative stress reaction and reduced neuronal apoptosis in T2DM mice suffering anesthesia/surgery.

The LTP outcome indicated that the mean of fEPSP slope was elevated remarkably in the hM4Di + CNO group after TBS compared to that in mCherry+CNO group (Figure 7J,K). We evaluated the cognitive function of both groups by performing the OLT and NORT. There was no significant difference for the percentage of exploration time toward the two objects in training phase during both NORT and OLT (Figure 7L,N). However, the percentage of exploration time for the novel object in NORT from hM4Di + CNO group mice was dramatically higher than that from mCherry+CNO group mice (Figure 7M). Consistently, the percentage of exploration time for the object in novel location in OLT from hM4Di + CNO group mice was also higher than that from mCherry+CNO group mice (Figure 7O). The above data showed that DREADDs improved functional synaptic plasticity and cognitive function in T2DM mice suffering anesthesia/surgery.

The above results suggested that chemogenetic inhibition of hippocampal glutamatergic neurons could directly prevent the occurrence of hyperexcitability and alleviate cognitive impairment in T2DM mice exposed to anesthesia/surgery.

## 4 DISCUSSION

In the present study, anesthesia/surgery was performed on wild‐type mice and type 2 diabetic mice around 3 months old rather than old mice. We found that young adult mice with type 2 diabetes but not wild‐type mice developed significant cognitive impairment after anesthesia/surgery, and these memory impairments were caused by hyperexcitability in the hippocampus. The results showed that type 2 diabetes enhanced the increased neuronal excitability induced by isoflurane anesthesia/surgery and caused cognitive impairment in young adult mice after tibial fracture surgery. Importantly, we also demonstrated the effect of hippocampal astrocytes GLT‐1 deficiency in postoperative memory deficits in type 2 diabetic mice. We found GLT‐1 deficiency alone couldn't lead to cognitive decline, but the lack of GLT‐1 meant that excitatory stimulation induced by anesthesia/surgery could not be dealt with and the subsequent increase in neuronal excitability was magnified. In other words, the neuronal hyperexcitability that led to POCD was caused by a combination of GLT‐1 deficiency and excitatory stimulation induced by anesthesia/surgery, which explained why POCD only occurred in T2DM + A/S mice. Overexpression of GLT‐1 could attenuate neuronal hyperexcitability and alleviate the above‐mentioned memory impairment. Finally, we proved that using chemogenetic means to specifically inhibit the excitability of hippocampal glutamatergic neurons could effectively reverse neuronal hyperexcitability and prevent corresponding cognitive impairment in T2DM mice suffering anesthesia/surgery. The consequences suggested that adult individuals who have type 2 diabetes may be more susceptible to POCD and provided a possible explanation for the clinically observed inter‐individual differences in the occurrence of cognitive dysfunction after anesthesia and surgery.

Postoperative cognitive dysfunction is a serious postoperative complication that will affect the life quality of patients and increase the risk of death, but its pathogenesis is still unclear, and there is no reasonable and effective intervention.^40^ It is generally believed that old age is an independent risk factor for postoperative cognitive dysfunction. Although postoperative cognitive impairment mostly occurs in elderly patients, clinical cases of POCD also often occur in young and middle‐aged patients. It is suggested that there should be other susceptible factors besides old age.^14^, ^41^

Many studies have explored diabetes‐related cognitive impairment, which is generally believed to occur at a later stage of the course of the disease^42^, ^43^ and verified that anesthesia/surgery can greatly aggravate this cognitive impairment.^44^ But here, we chose the young adult mice in early type 2 diabetic without obvious cognitive‐related behavioral changes. In addition, previous studies have reported significant cognitive impairment in elderly mice undergoing general anesthesia and surgery, while the present research has found that healthy adult mice did not exhibit significant cognitive impairment after anesthesia/surgery. However, significant cognitive impairment occurs after anesthesia/surgery in these early T2DM adult mice instead of healthy adult mice. This is a very interesting phenomenon, which suggests that type 2 diabetes may be another susceptible factor besides old age. The current data also indicated that hippocampal glutamatergic neurons were activated after anesthesia/surgery, and reduced GLT‐1 expression in hippocampal astrocytes of type 2 diabetic mice significantly amplified the increase in glutamatergic neuron excitability induced by anesthesia/surgery and triggered POCD in early T2DM adult mice. This was demonstrated by the results that overexpression of GLT‐1 in hippocampal astrocytes or inhibition of glutamatergic neuron excitability using chemogenetic means can effectively prevent or alleviate this hippocampus‐dependent cognitive impairment induced by hyperexcitability in T2DM mice suffering anesthesia/surgery.

Isoflurane is one of the most commonly used inhalational anesthetics for general anesthesia. Previous studies have shown that the excitability of neurons in mice increases after awakening under general anesthesia, especially with inhaled anesthetics.^45^, ^46^ For a long time, the research on POCD has mainly focused on neuroinflammation, synaptic plastic injury, and postoperative stress.^34^, ^47^ The overexcitation of neurons has not received much attention. Existing studies have shown that excessive excitability can easily lead to excitotoxicity,^48^ which plays an important role in acute brain injury of epilepsy, stroke, and neurodegenerative diseases.^49^, ^50^, ^51^ Glutamatergic neurons in hippocampal CA1 region are closely related to learning ability and memory formation.^52^ Previous studies have shown that glutamatergic neurons excessive activation in the CA1 region will lead to hippocampal LTP weakness and affect the cognitive function of mice.^53^ We found that 3 h after anesthesia surgery, the glutamatergic neurons excitability in normal mice and T2DM mice was enhanced, while the excitability of GABAergic neurons did not change significantly, which was consistent with previous research. At the same time, we found that the increase in glutamatergic neuron excitability in T2DM mice was significantly greater than that in normal mice after anesthesia/surgery. These results suggest that anesthesia/surgery may cause cognitive dysfunction by inducing neuronal hyperexcitability in T2DM mice instead of healthy adult mice.

Hyperexcitiabililty can promote oxidative stress and neuronal apoptosis.^7^, ^48^ In the present study, it was found that T2DM mice developed significant mitochondrial oxidative stress, which was mainly manifested as down‐regulation of SOD2 expression and reduced mitochondrial membrane potential levels after undergoing anesthesia/surgery. Oxidative stress is an imbalance within an organism, mainly due to the excessive production of free radicals, which exceeds the scavenging capacity of the antioxidant system.^54^ This imbalance can have many negative effects on the body and is considered an important factor in cognitive dysfunction.^55^ Excessive free radicals can damage the membrane structure, mitochondria, and organelle functions of hippocampal neuron. The damage of free radicals to mitochondria is particularly significant, because mitochondria are the main energy production center in cells, and mitochondrial oxidative stress can lead to cellular energy metabolism disorders and increased cell death.^56^ Antioxidants are thought to potentially help slow down the process of oxidative stress, thereby alleviating or delaying the progression of cognitive dysfunction.^57^ However, relevant clinical research results are inconsistent, and more in‐depth research is needed to determine its exact effect and optimal timing of use.^58^ Oxidative stress is one of the important factors in cognitive dysfunction, and a thorough understanding of its mechanisms and effects is of great significance.

In addition, the proportion of cell apoptosis by Tunel staining was significantly increased in the hippocampal CA1 area of T2DM mice suffering anesthesia/surgery. Subsequently, we injected chemical genetic inhibitory viruses into the hippocampus to specifically reduce the activity of hippocampal glutamatergic neurons. The results showed that prevention of the hyperexcitability in hippocampal CA1 glutamatergic neurons markedly weakened mitochondrial oxidative stress, reduced neuronal apoptosis and promoted the recovery of long‐term potentiation and cognitive function in T2DM mice suffering anesthesia/surgery. The results further demonstrated that hippocampal mitochondrial oxidative stress and neuronal apoptosis induced by neuronal hyperexcitability may contribute to the occurrence of POCD in T2DM mice after anesthesia/surgery.

GLT‐1, mainly expressed in astrocytes, plays an irreplaceable role in scavenging extracellular glutamate and preventing neuronal excitotoxicity.^26^, ^59^ Its abnormal expression is closely related to addiction, epilepsy, and Alzheimer's disease, mainly because it significantly affects the excitability of neurons.^60^, ^61^, ^62^ The overexpression of GLT‐1 in astrocytes can antagonize the overexcitation of neurons.^63^ Existing studies have shown that obesity and high‐fat diet affect the expression and function of GLT‐1 in astrocytes.^31^, ^64^ In our study, we observed that type 2 diabetes suppressed the expression of GLT‐1, and magnified increased excitability of hippocampal glutamatergic neurons induced by anesthesia/surgery. Overexpression of GLT‐1 in the hippocampal astrocytes significantly attenuated the hyperexcitability of hippocampal glutamatergic neurons, reduced oxidative stress and neuronal apoptosis, improved LTP and cognitive function in T2DM mice suffering anesthesia/surgery, which suggested that the excitotoxic injuries of glutamatergic neurons in hippocampus induced by reduced expression of GLT‐1 in astrocytes may trigger the cognitive dysfunction in T2DM mice after isoflurane anesthesia/surgery.

In clinical practice, the proportion of elderly people among all the diabetic patients is very considerable.^42^, ^65^ First, it is very important and necessary to clarify whether the diabetes factor and the old age factor can synergistically promote the occurrence of POCD in the future study, since old age is an independent risk factor for POCD.^6^, ^31^ Second, although the key role of hippocampal astrocyte GLT‐1 in POCD has been confirmed in T2DM mice after anesthesia/surgery, further evidence is needed to clarify the mechanism by which high glucose or insulin resistance induces a decrease of GLT‐1. In addition, the β‐lactam antibiotic ceftriaxone (CEF) is a potent GLT‐1 translational activator^66^ and may be a promising drug for preventing and treating POCD especially in patients with diabetes, which remains to be further investigated in the future.

## 5 CONCLUSION

**FIGURE 8:**
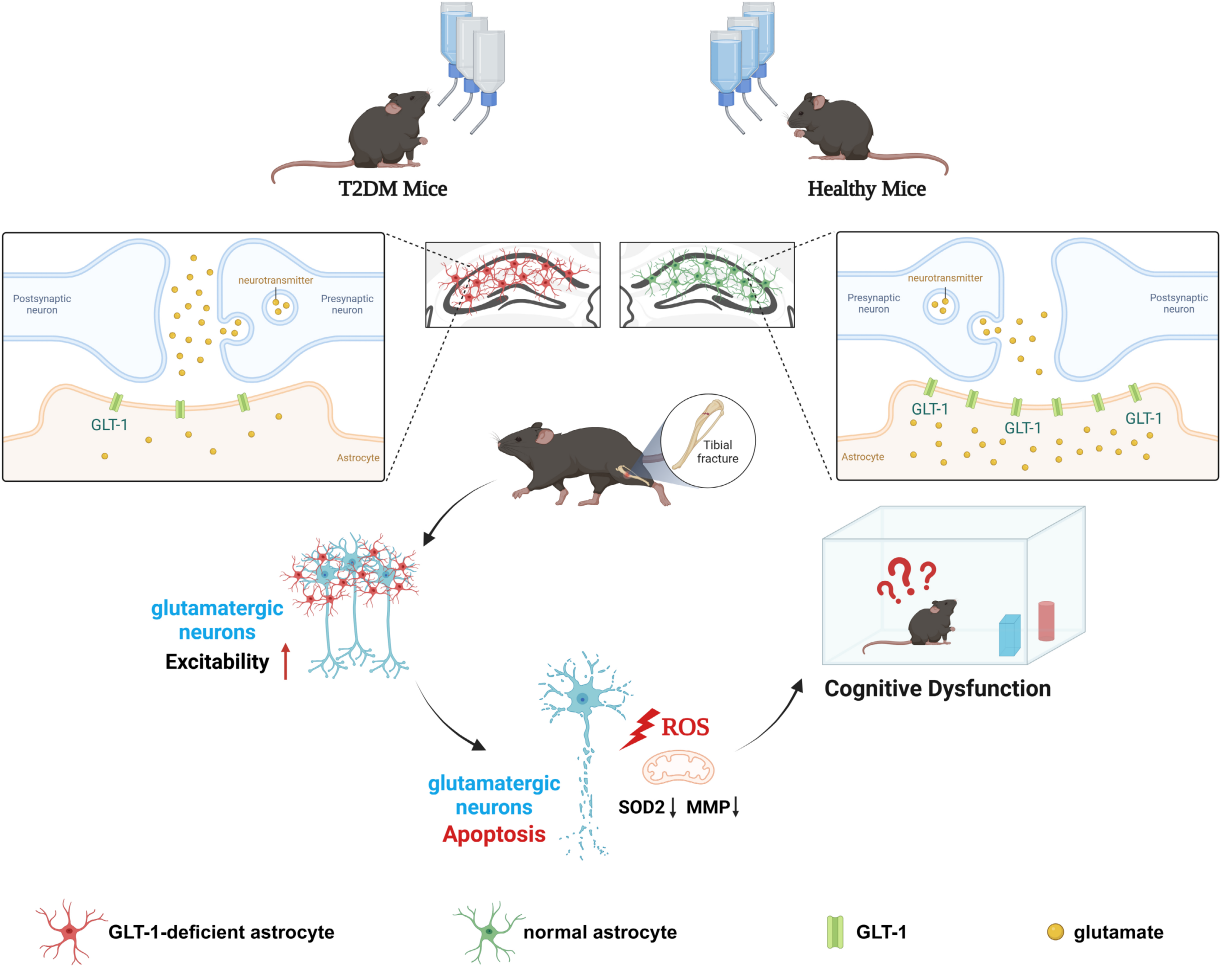
The schematic diagram shows that GLT‐1 plays a key role in T2DM‐induced cognitive impairment in mice after anesthesia/surgery. Due to the downregulation of GLT‐1 expression in hippocampal astrocytes caused by diabetes, the increased excitability of glutamatergic neurons induced by anesthesia/surgery was amplified in the local hippocampal, which led to oxidative stress, apoptosis and finally cognitive dysfunction.

In summary, the present study found that diabetes may be associated with an increased likelihood of cognitive decline after anesthesia/surgery. These findings suggest that GLT‐1 downregulation in type 2 diabetes can enhance the increase in hippocampal glutamatergic neuron excitability induced by anesthesia/surgery, which contributes to the development of cognitive impairment in T2DM mice suffering surgery under isoflurane anesthesia (Figure 8). Attenuation of hippocampal excitotoxicity by increasing GLT‐1 in hippocampal astrocytes could effectively prevent postoperative cognitive dysfunction in T2DM mice. This study provides an important insight into the mechanism by which patients with diabetes may be susceptible to postoperative cognitive dysfunction. In the future studies, it is essential to clarify whether type 2 diabetes is an independent risk factor for POCD in clinical settings for the purpose of ensuring intraoperative safety and reducing the risk of perioperative complications in diabetics.

## AUTHOR CONTRIBUTIONS

Under the guidance of YQ Wu, CH Zhou, and Q Liu, XH Jiao, J Wan, and WF Wu completed the design. All the other authors performed the experiments. All the authors participated in data collection and statistical analysis. The manuscript was written by XH Jiao and YQ Wu. All the authors have read and approved the final manuscript. YQ Wu secured funding for the project.

## CONFLICT OF INTEREST STATEMENT

The authors declare no competing interests.

## Supporting information


Figure S1.

File S1.

## Data Availability

The data that support the findings of this study are available from the corresponding author upon reasonable request.
