# Supplementary figures and images for: GLT‐1 downregulation in hippocampal astrocytes induced by type 2 diabetes contributes to postoperative cognitive dysfunction in adult mice
Source: CNS Neurosci Ther. 2024 Sep 1;30(9):e70024. doi: 10.1111/cns.70024 (PMC11366448; doi:10.1111/cns.70024)

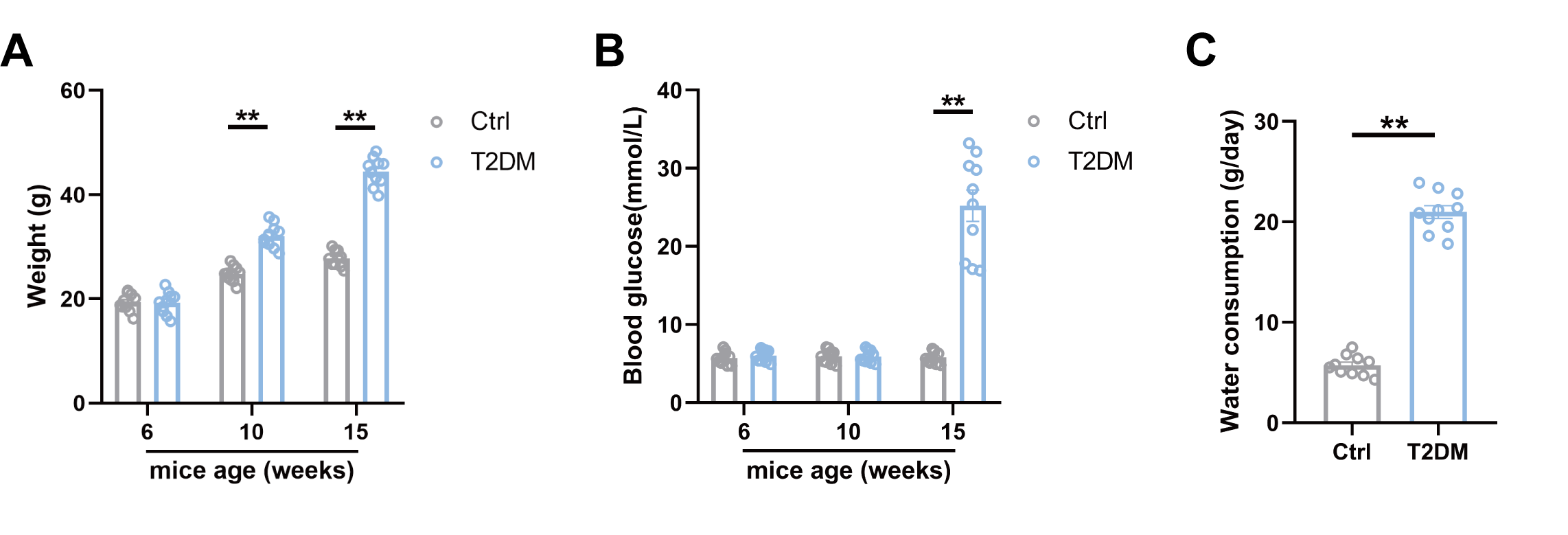

Supplement: Supplementary file 1 — Figure S1. [file CNS-30-e70024-s002.tif]
