# Supplementary material for: GLT‐1 downregulation in hippocampal astrocytes induced by type 2 diabetes contributes to postoperative cognitive dysfunction in adult mice
Source: CNS Neurosci Ther. 2024 Sep 1;30(9):e70024. doi: 10.1111/cns.70024 (PMC11366448; doi:10.1111/cns.70024)

The lanes of the unedited blot that appear in the cropped image in the manuscript have been highlighted in red box

Full unedited blot for Fig.2A

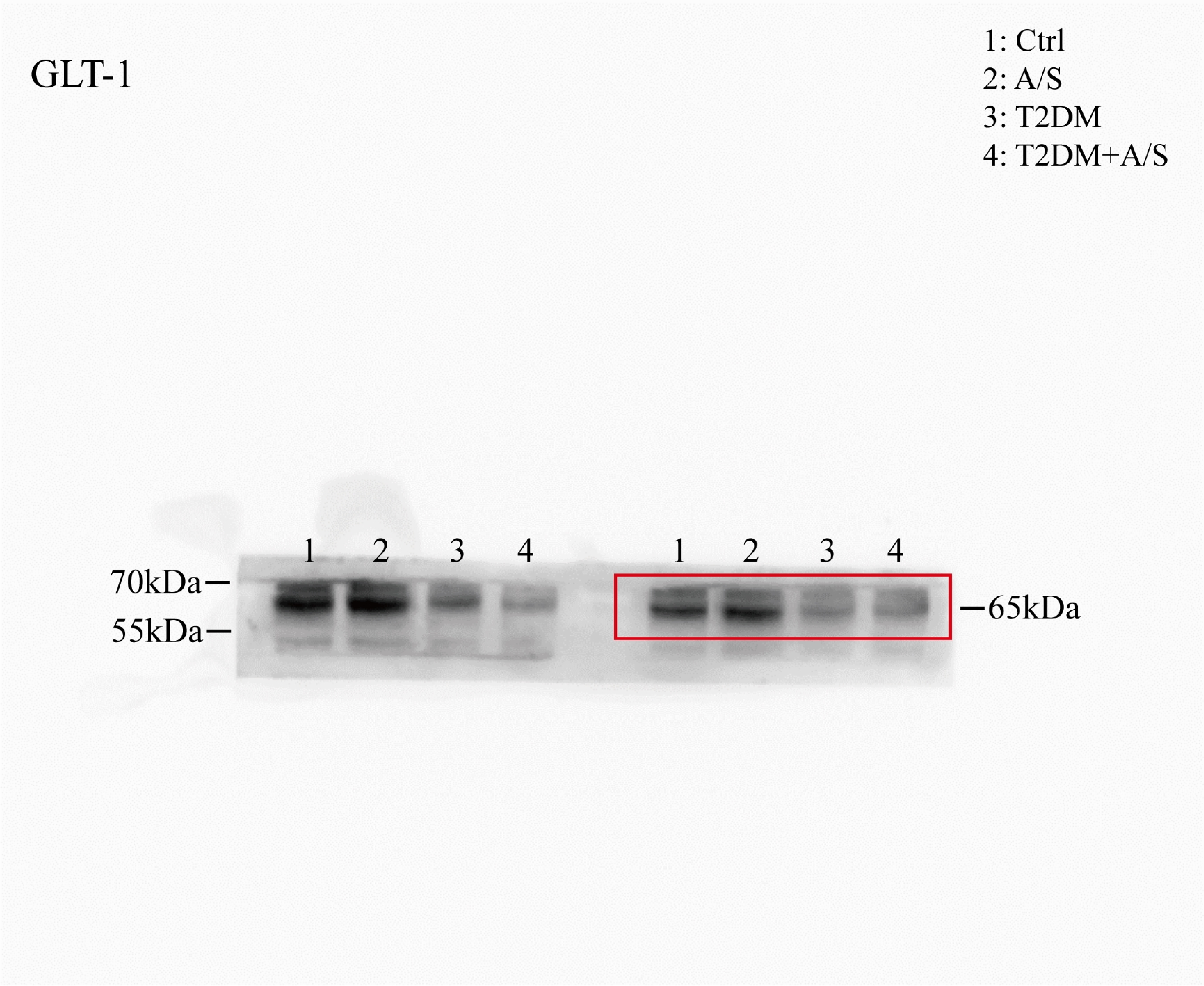

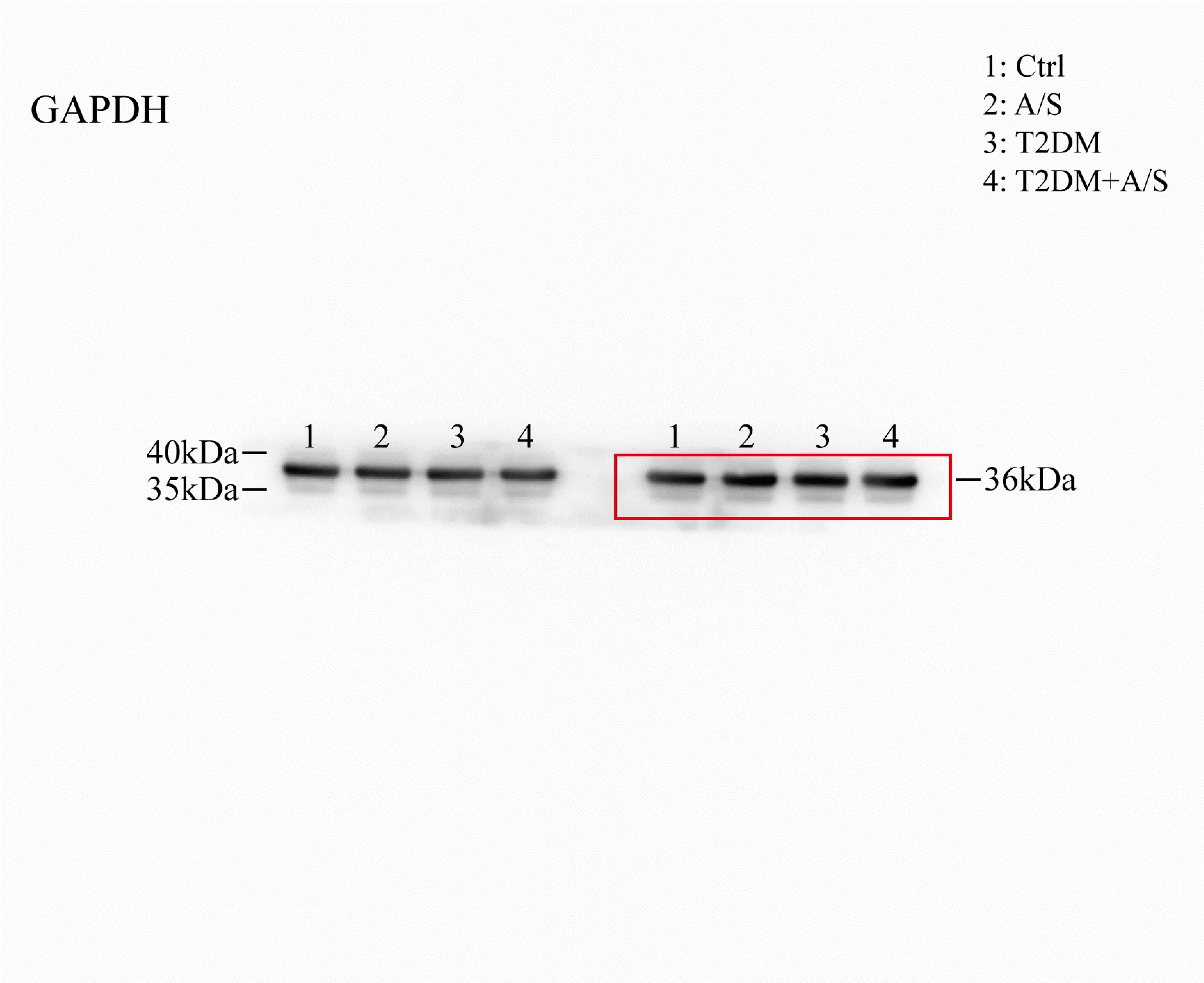

Full unedited blot for Fig.4A

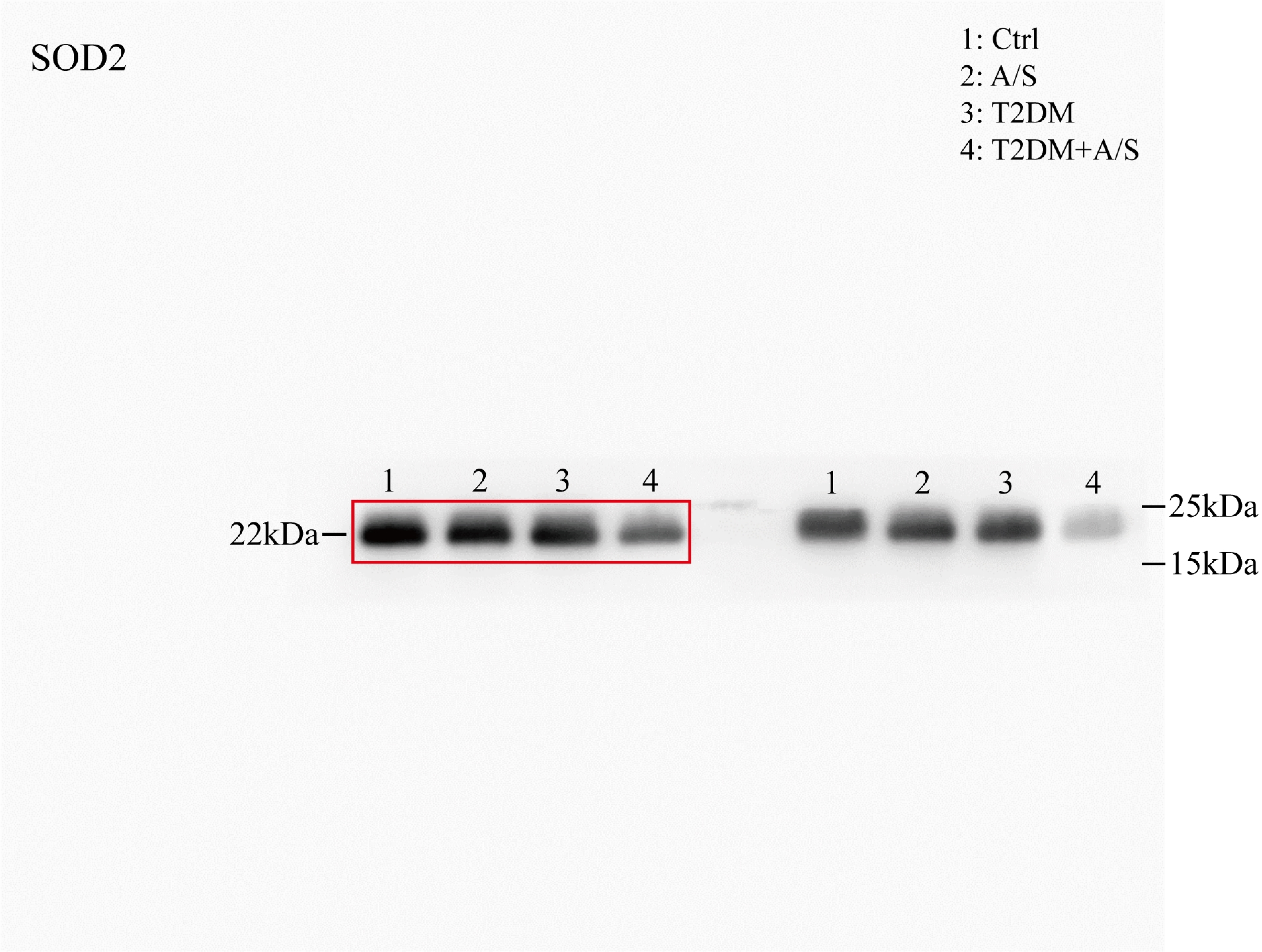

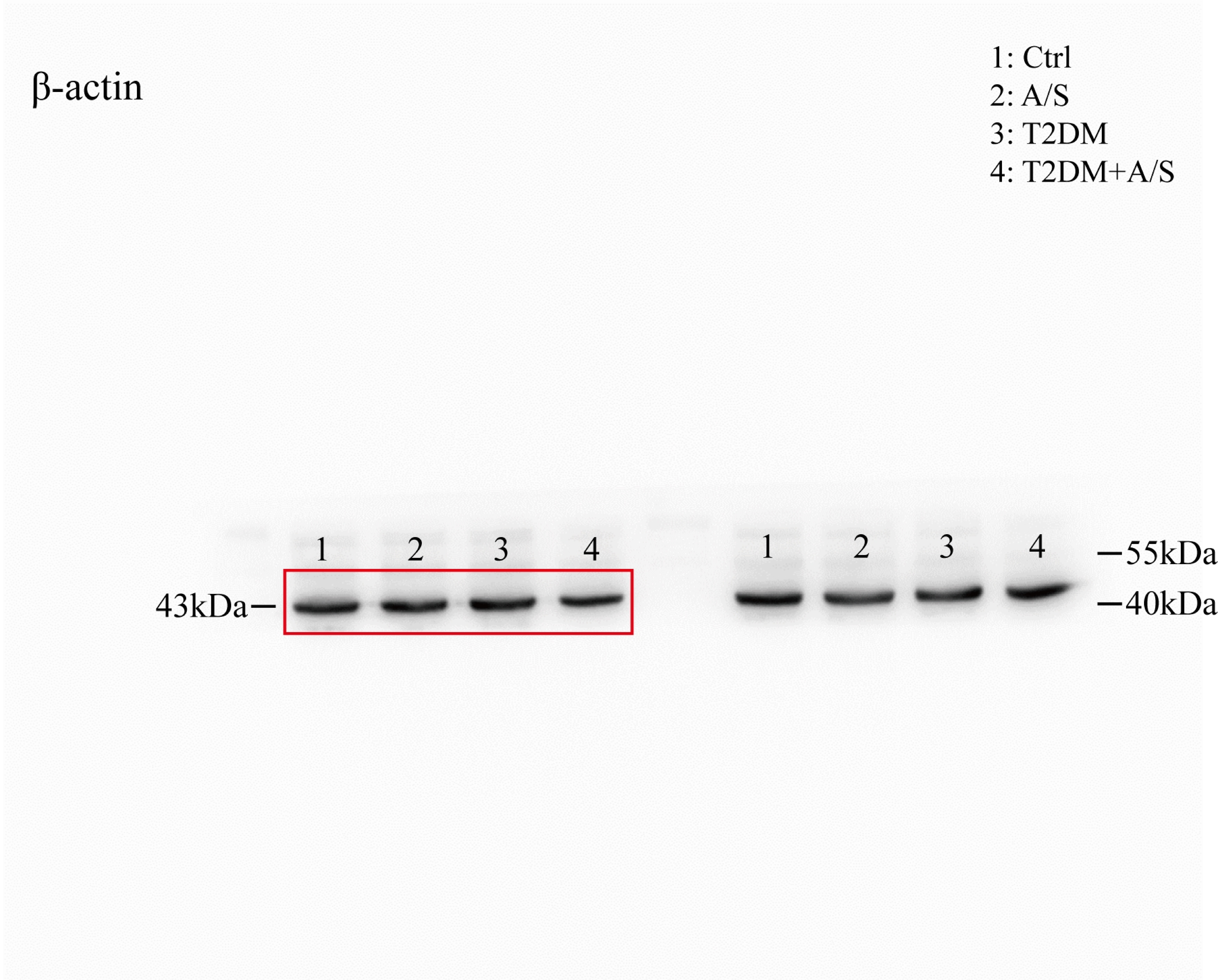

Full unedited blot for Fig.5G

GLT-1

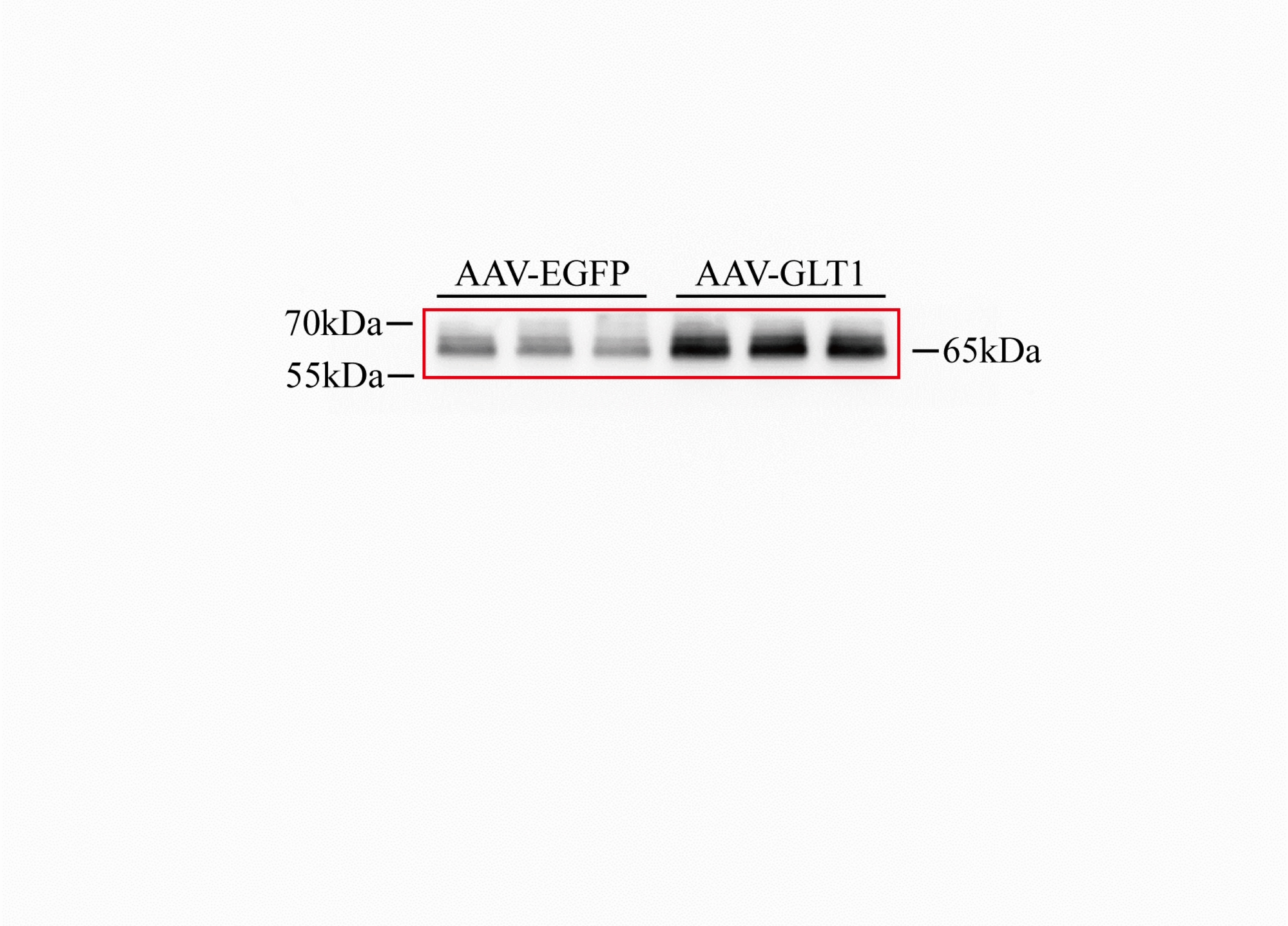

GAPDH

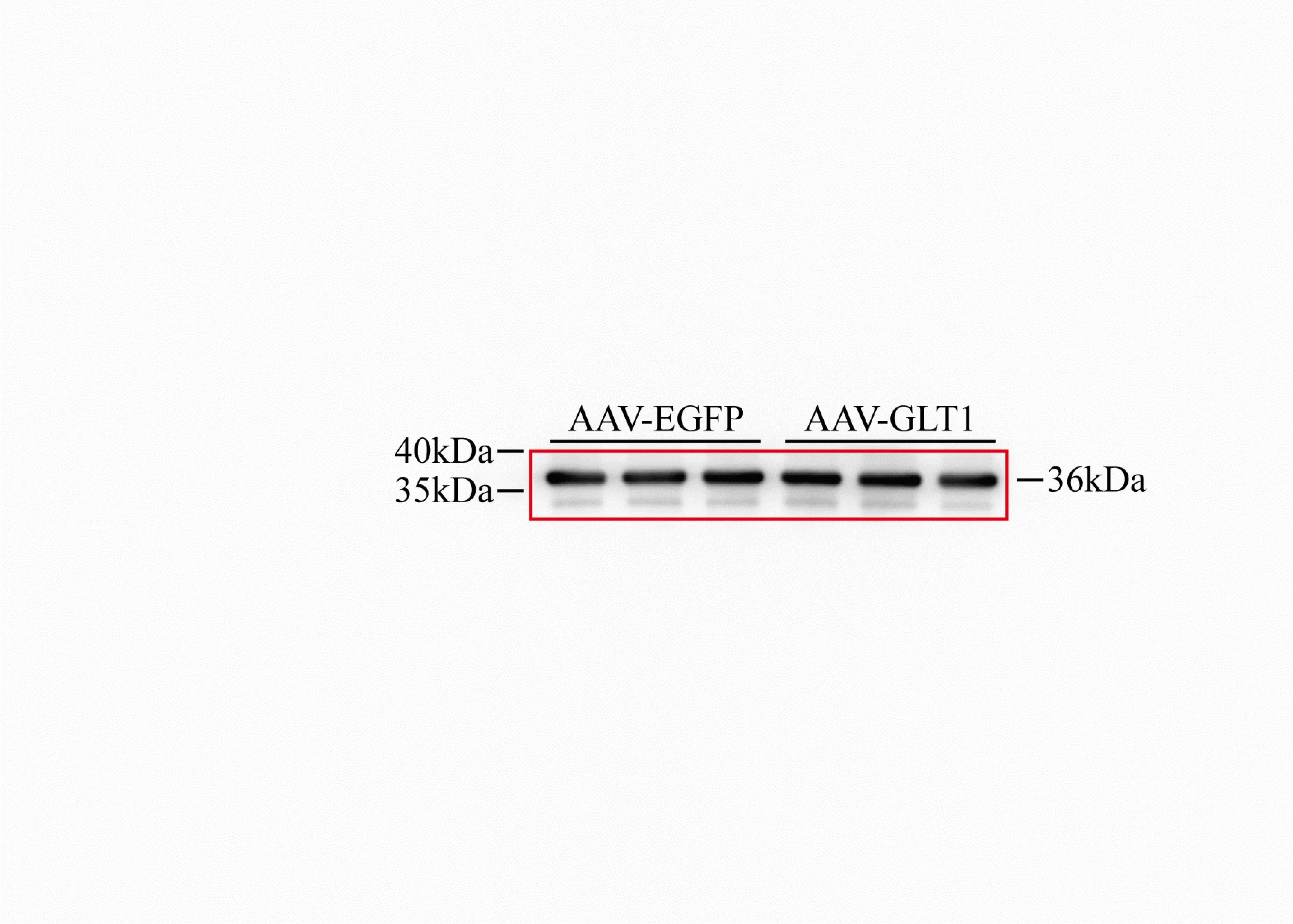

Full unedited blot for Fig.6C

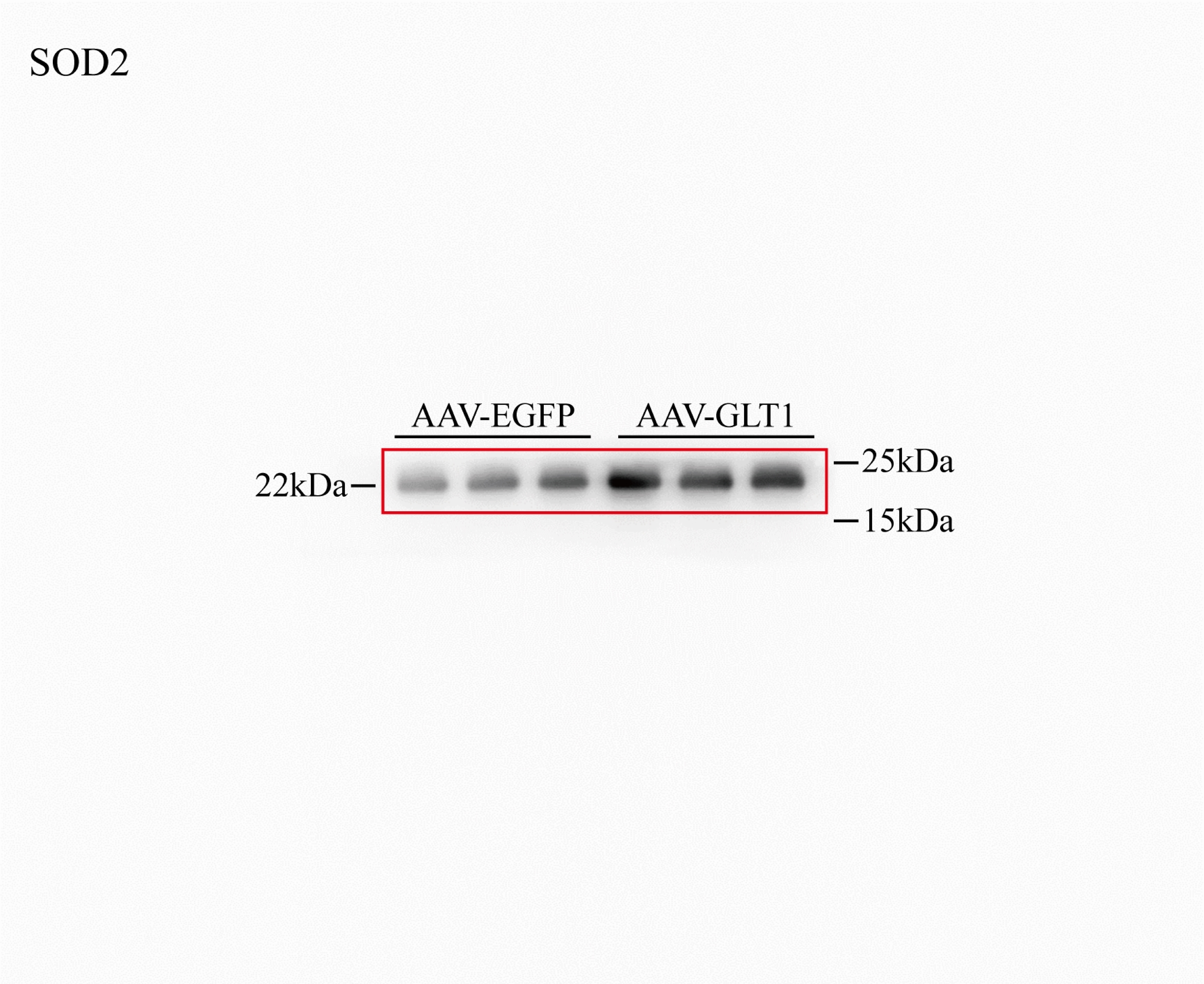

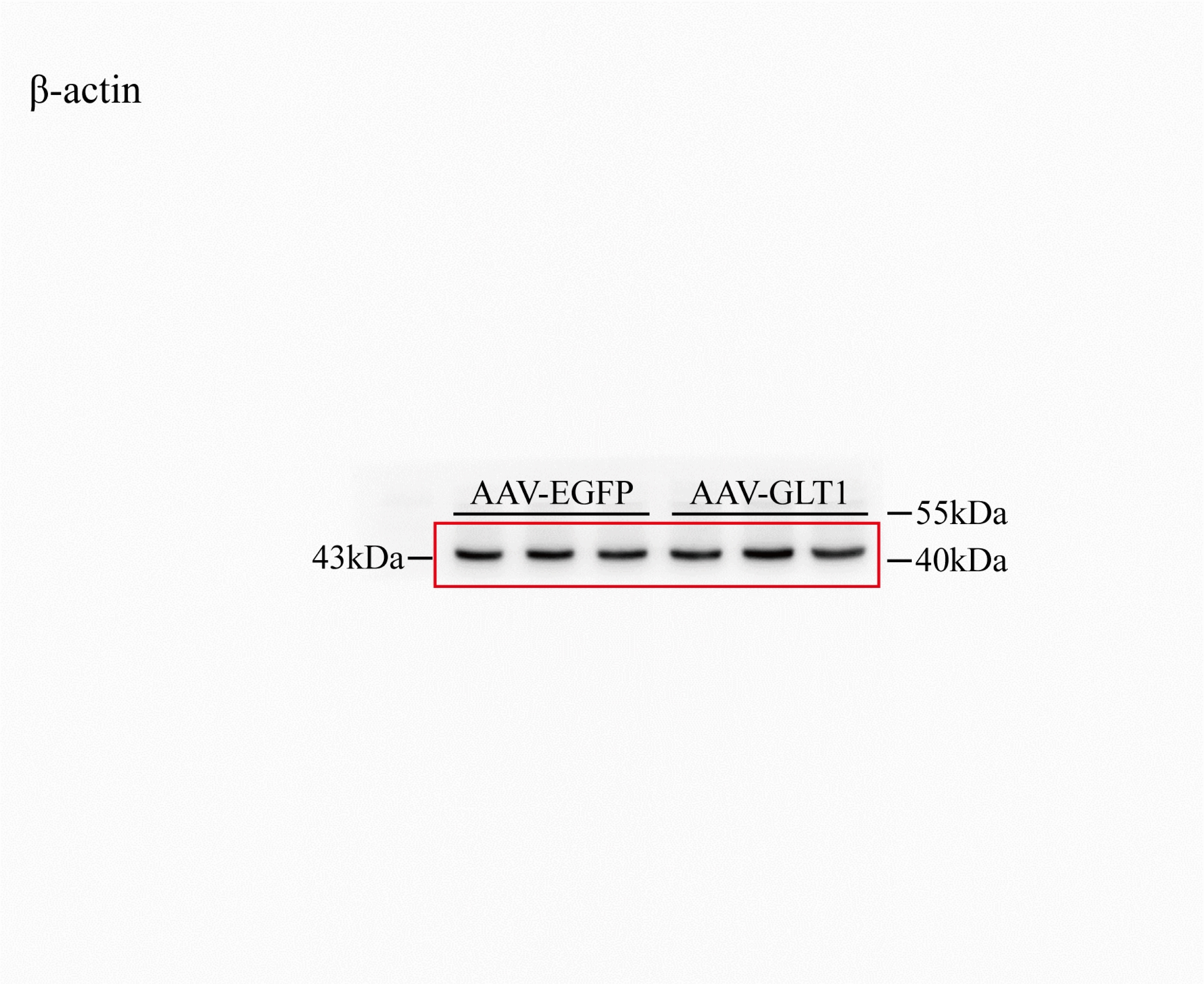

Full unedited blot for Fig.7E

SOD2

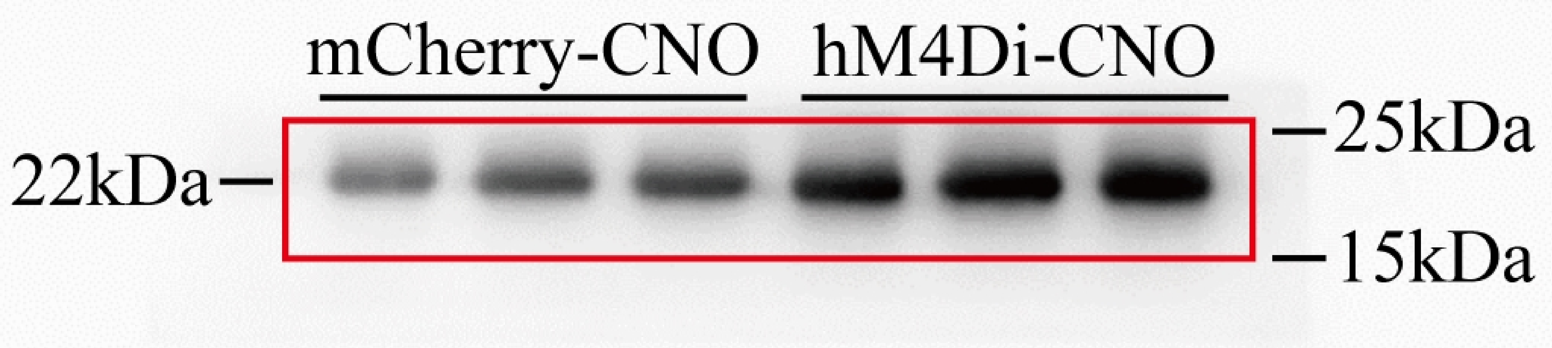

β-actin

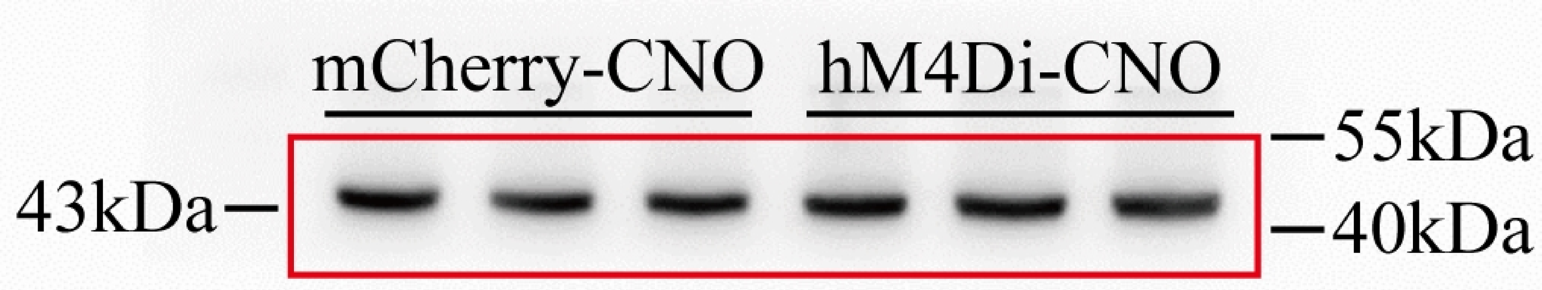

Supplement: Supplementary file 2 — File S1. [file CNS-30-e70024-s001.pdf]
